# Genetic synergy between *Acinetobacter baumannii* undecaprenyl phosphate biosynthesis and the Mla system impacts cell envelope and antimicrobial resistance

**DOI:** 10.1128/mbio.02804-23

**Published:** 2024-02-16

**Authors:** Hannah R. Noel, Sowmya Keerthi, Xiaomei Ren, Jonathan D. Winkelman, Jerry M. Troutman, Lauren D. Palmer

**Affiliations:** 1 Department of Microbiology and Immunology, University of Illinois Chicago, Chicago, Illinois, USA; 2 Department of Chemistry, University of North Carolina Charlotte, Charlotte, North Carolina, USA; 3 Trestle LLC, Milwaukee, Wisconsin, USA; The University of Kansas Medical Center, Kansas City, Kansas, USA

**Keywords:** *Acinetobacter*, antibiotic resistance, membrane stress, isoprenoid, Und-P, maintenance of lipid asymmetry, lipooligosaccharide, LOS, capsule

## Abstract

*Acinetobacter baumannii* is a Gram-negative bacterial pathogen that poses a major health concern due to increasing multidrug resistance. The Gram-negative cell envelope is a key barrier to antimicrobial entry and includes an inner and outer membrane. The maintenance of lipid asymmetry (Mla) system is the main homeostatic mechanism by which Gram-negative bacteria maintain outer membrane asymmetry. Loss of the Mla system in *A. baumannii* results in attenuated virulence and increased susceptibility to membrane stressors and some antibiotics. We recently reported two strain variants of the *A. baumannii* type strain ATCC 17978: 17978VU and 17978UN. Here, ∆*mlaF* mutants in the two ATCC 17978 strains display different phenotypes for membrane stress resistance, antibiotic resistance, and pathogenicity in a murine pneumonia model. Although allele differences in *obgE* were previously reported to synergize with ∆*mlaF* to affect growth and stringent response, *obgE* alleles do not affect membrane stress resistance. Instead, a single-nucleotide polymorphism (SNP) in the essential gene encoding undecaprenyl pyrophosphate (Und-PP) synthase, *uppS*, results in decreased enzymatic rate and decrease in total Und-P levels in 17978UN compared to 17978VU. The UppS^UN^ variant synergizes with ∆*mlaF* to reduce capsule and lipooligosaccharide (LOS) levels, increase susceptibility to membrane stress and antibiotics, and reduce persistence in a mouse lung infection. Und-P is a lipid glycan carrier required for the biosynthesis of *A. baumannii* capsule, cell wall, and glycoproteins. These findings uncover synergy between Und-P and the Mla system in maintaining the *A. baumannii* cell envelope and antibiotic resistance.

**IMPORTANCE:**

*Acinetobacter baumannii* is a critical threat to global public health due to its multidrug resistance and persistence in hospital settings. Therefore, novel therapeutic approaches are urgently needed. We report that a defective undecaprenyl pyrophosphate synthase (UppS) paired with a perturbed Mla system leads to synthetically sick cells that are more susceptible to clinically relevant antibiotics and show reduced virulence in a lung infection model. These results suggest that targeting UppS or undecaprenyl species and the Mla system may resensitize *A. baumannii* to antibiotics in combination therapies. This work uncovers a previously unknown synergistic relationship in cellular envelope homeostasis that could be leveraged for use in combination therapy against *A. baumannii*.

## INTRODUCTION

*Acinetobacter baumannii* is a Gram-negative bacterial pathogen that is a major cause of healthcare-associated infections. Clinical isolates of *A. baumannii* demonstrate resistance to first line antibiotics such as meropenem and last resort antibiotics such as colistin (1). The World Health Organization and the Centers for Disease Control name *A. baumannii* as an urgent threat, calling for the development of novel antimicrobials (2–4). *A. baumannii* has multiple intrinsic mechanisms for resisting antibiotics and host stressors. The first line of defense is the cellular envelope including capsule, a peptidoglycan cell wall, and a dual-membrane system conserved among Gram-negative bacteria (5, 6). The inner membrane (IM) and outer membrane (OM) protect Gram-negative bacteria from environmental stress (7, 8). The Gram-negative OM is asymmetric with phospholipids composing the inner leaflet and lipopolysaccharides (LPS) or lipooligosaccharides (LOS) composing the outer leaflet. *A. baumannii* does not encode the gene required for O-antigen elaboration, resulting in LOS rather than LPS (9, 10). Additionally, *A. baumannii* is able to survive without LOS, whereas in most Gram-negative bacteria, LPS is essential (11–13). In all Gram-negative bacteria, the asymmetric bilayer of the OM is critical for resistance to membrane stressors and antimicrobials (14, 15).

The maintenance of lipid asymmetry (Mla) system is thought to be the primary homeostatic mechanism to maintain OM lipid asymmetry by removing mislocalized phospholipids from the outer leaflet of the OM (16). MlaBDEF forms an IM ATP-binding cassette (ABC) transporter with ATPase activity, MlaC is a periplasmic protein, and MlaA is an OM lipoprotein that forms a complex with OmpC/F (16–19,20). While the Mla system is not required for growth in lysogeny broth, bacteria lacking a functional Mla system are more sensitive to the membrane stress SDS/EDTA (16, 17, 21–23). Inactivating *mlaF* or *mlaC* in multiple species results in the loss of function of the Mla system and increased sensitivity to membrane stressors, antibiotics, and the host (21, 24–26). Additionally, dominant negative mutations in *mlaA* (mlaA*) have been shown to increase outer membrane permeability and susceptibility to erythromycin and rifampicin in *Escherichia coli* (27). The Mla system is necessary for the virulence of multiple pathogenic species including *Shigella flexneri*, *Burkholderia pseudomallei*, and *Pseudomonas aeruginosa* (28–32). By contrast, mutations in the Mla system have been shown to increase virulence in *E. coli* and *Neisseria gonorrhoeae* (33, 34). In summary, the Mla system is critical for Gram-negative outer membrane maintenance, stress resistance, and virulence.

While the directionality of lipid transport by the Mla system has been debated, the preponderance of evidence supports a retrograde transport model of phospholipid movement from the OM to the IM (35–37). Genetic evidence from *E. coli, A. baumannii,* and chloroplasts supports a retrograde transport model in which phospholipids are removed from the outer leaflet of the OM and transported to the IM (16, 21, 27, 38–41). Additionally, crystallographic and cryo-EM structural data from *Klebsiella pneumoniae*, *Serratia marcescens,* and *E. coli* support a model in which phospholipids are removed from the OM by the MlaA-OmpF complex and transported toward MlaBDEF based on the orientation of MlaA-OmpF in the OM (17, 42–44). Recent studies identifying AsmA-like proteins facilitating anterograde lipid transport machinery in *E. coli* further support the retrograde transport model for the Mla system (45, 46). By contrast, *in vitro* work in *E. coli* as well as cryo-EM, molecular dynamics, and pulse-chase studies in *A. baumannii* described an anterograde transport model where newly synthesized phospholipids are transported to the inner leaflet of the OM (19, 24, 47, 48). However, Mann et al., whose structural work in *A. baumannii* supports an anterograde lipid transport model, speculate that nucleotide state or solubilization approach of the MlaBDEF studies may explain the differences in conclusions (48). Recent structures of MlaBDEF from *E. coli* and *A. baumannii* and *E. coli* MlaC in complex with MlaA or MlaD were solved and provide mechanistic insight on lipid binding but do not provide clear evidence for either direction of lipid transport (22, 49, 50).

In *A. baumannii*, the Mla system synergizes with essential pathways to promote growth. A previous study reported synergy between the Mla system and the essential GTPase ObgE in promoting Δ*mlaF* growth and stringent response (51, 52). We previously reported a suppressor mutation in the isoprenoid biosynthetic pathway that restored resistance of an *A. baumannii* Δ*mlaF* mutant to membrane stress, some antibiotics, and host stressors (21). The suppressor is an IS*Aba11* transposition in the 5′ untranslated region of *ispB* that results in *ispB* downregulation (21). We hypothesized that the downregulation of *ispB* increased flux of the branchpoint substrate farnesyl-pyrophosphate (FPP) to undecaprenyl pyrophosphate (Und-PP) synthase, UppS. Und-PP is a precursor to the essential glycan carrier undecaprenyl phosphate (Und-P), also known as bactoprenol phosphate (BP), that transfers glycans across the plasma membrane for biosynthesis of peptidoglycan, capsular polysaccharides, and the O-antigen in LPS-producing bacteria (53). This finding suggested a role for Und-P biosynthesis in membrane stress resistance in the absence of the Mla system in *A. baumannii*. Thus, multiple reports have identified synergy between the Mla system and other pathways in *A. baumannii*.

Recently, we reported two variants of the commonly used laboratory strain *A. baumannii* 17978 distributed by ATCC (54). These variants, *A. baumannii* ATCC 17978VU and 17978UN, have distinct genotypes with six protein-encoding single-nucleotide polymorphisms (SNPs) and a 44 kb accessory locus (AbaAL44) that is present in 17978UN but absent in 17978VU (54). The protein-coding SNPs encode variants of the lipooligosaccharide transporter LptD and the essential proteins ObgE and UppS (55). Our previous work used isogenic ∆*mlaF* strains in the 17978UN (AbaAL44^+^) background (21). Upon reconstructing ∆*mlaF* strains in the *A. baumannii* ATCC 17978VU strain background, we discovered that the 17978VU *∆mlaF* mutant was more resistant to SDS/EDTA membrane stress than the 17978UN *∆mlaF* mutant. Here, we describe genetic synergy between the maintenance of lipid asymmetry and Und-P biosynthesis uncovered through genetic dissection and comparison of ATCC 17978VU and 17978UN ∆*mlaF* strains. These findings suggest an underlying relationship between the Mla system and undecaprenyl biosynthesis in *A. baumannii* that function together to maintain LOS abundance and promote membrane stress resistance, virulence, and antimicrobial resistance.

## RESULTS

### UppS synergizes with the Mla system under membrane stress

**Fig 1:**
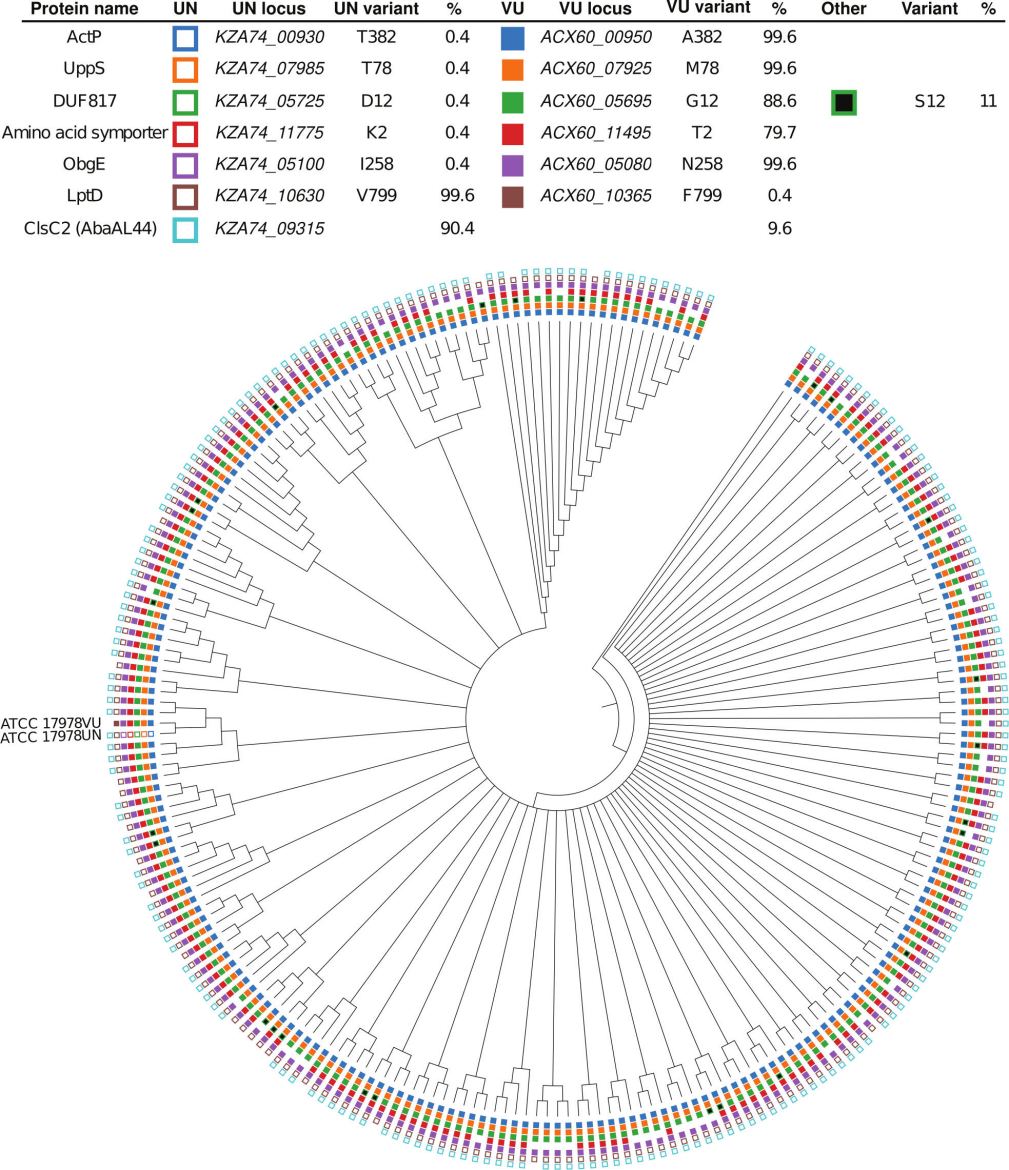
Phylogenetic tree of *Acinetobacter baumannii* strains depicting prevalence of variants encoded by ATCC 17978VU and 17978UN. Filled, non-black boxes indicate the presence of a protein variant identical to *A. baumannii* ATCC 17978VU, while unfilled boxes denote the presence of the *A. baumannii* ATCC 17978UN variant. The absence of a box indicates that a predicted ortholog was not found in the genome. Black boxes indicate the presence of a variant different from both the 17978VU and 17978UN strains. The presence of ClsC2, indicated by an unfilled box of the 17978UN strain, is representative of the presence of the 44 kb accessory locus AbaAL44.

**Fig 2:**
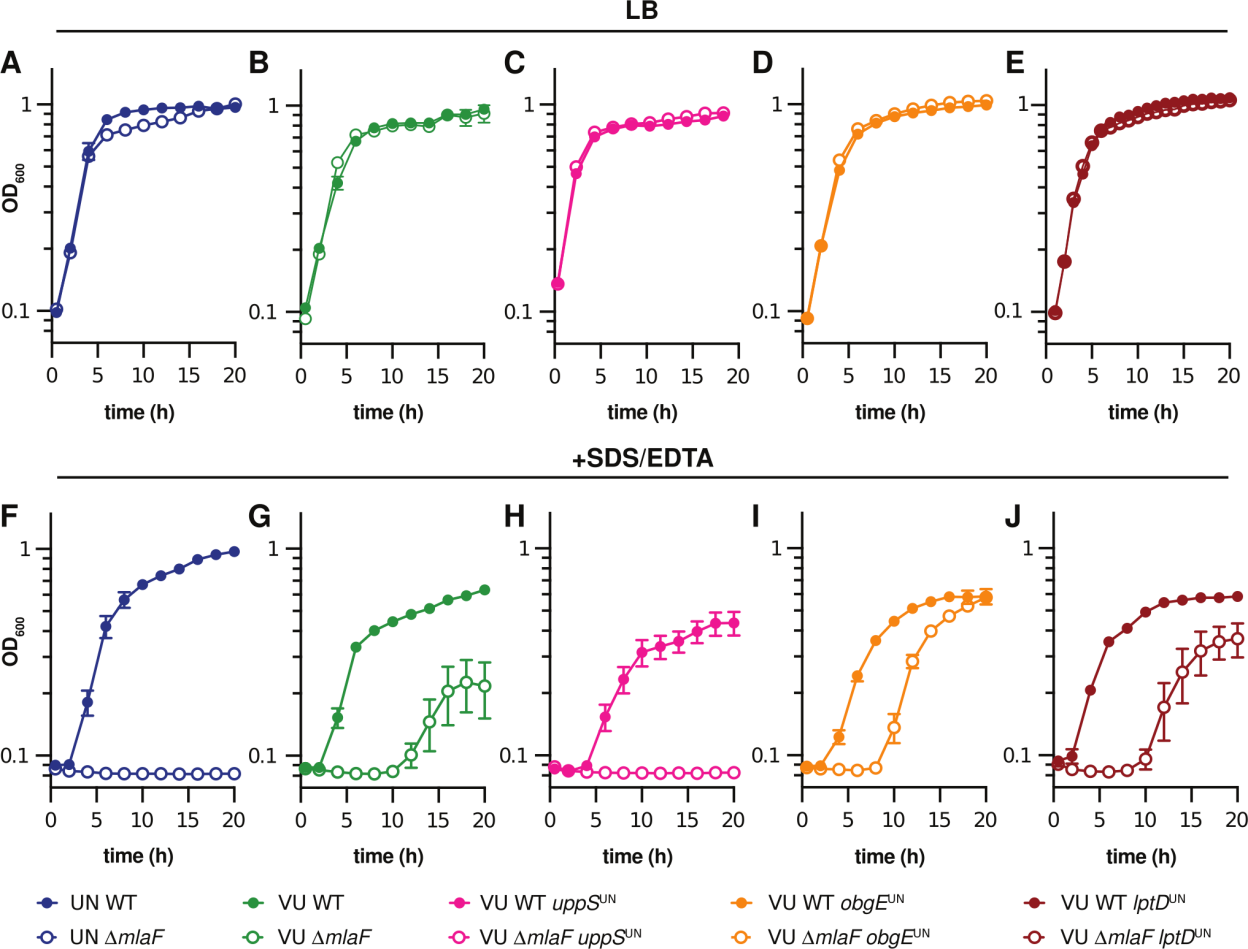
UppS^UN^ protein variant results in increased membrane stress sensitivity in *A. baumannii* ATCC 17978 ∆*mlaF* mutants. (A–E) 17978UN and 17978VU wild-type, Δ*mlaF,* and isogenic mutant strains were grown in LB. (F–J) 17978UN and 17978VU wild-type, Δ*mlaF,* and isogenic mutant strains were grown in LB with 0.01% SDS and 0.175 mM EDTA. Data are means ± SEM, *n* = 3.

The 17978VU and 17978UN strain variants of ATCC 17978 contain SNPs in multiple protein coding genes (54). We first assessed the prevalence of the 17978VU and 17978UN alleles across *A. baumannii* genomes. A set of 5945 genomes from NCBI were de-duplicated to remove near-clonal lineages and the predicted proteomes of the resulting 230 genomes were analyzed by OrthoFinder to deduce orthologues within *A. baumannii* (56, 57). The 17978UN predicted protein variants were more common for LptD (99%) and ClsC2 (indicative of the presence of the AbaAL44 cluster; 90%) (Fig. 1). The 17978VU predicted protein variants were more common for ObgE (99%), UppS (99%), ActP (99%), the amino acid symporter ACX60_11495 (79%), and DUF817 (89%) (Fig. 1). Thus, both 17978VU and 17978UN contain alleles representing the majority of published *A. baumannii* genomes. Next, the ∆*mlaF*::Kn (∆*mlaF*) mutation was reconstructed in *A. baumannii* ATCC 17978VU and SDS/EDTA membrane stress resistance was compared in the 17978VU (AbaAL44^−^) and 17978UN (AbaAL44^+^) strain backgrounds. Neither the wild-type strains nor the Δ*mlaF* mutants demonstrated a growth defect in lysogeny broth (LB) regardless of strain background (Fig. 2A and B). However, in the presence of SDS/EDTA membrane stress, the 17978UN Δ*mlaF* mutant exhibited a greater growth defect than the 17978VU Δ*mlaF* mutant (Fig. 2F and G), suggesting synergy between *mlaF* and genetic differences between the strains. Therefore, we reasoned that the closely related strain variants ATCC 17978VU and 17978UN could be used as a tool to uncover this synergy.

To determine the genetic cause behind the differences in membrane stress resistance between ATCC 17978VU and 17978UN Δ*mlaF* strains, point mutants were constructed for three candidate genes: *obgE*, *lptD*, and *uppS*. Candidate genes were chosen based on literature findings and known function. First, Powers et al. previously reported that *obgE* alleles in 17978 strains maintained at University of Washington (UW) and University of Georgia (UGA) synergized with *mlaF* to affect growth and stringent response. Specifically, the ∆*mlaF* strain with ObgE^I258^ (ObgE^UN^) had defects in LB growth and stringent response compared to the ∆*mlaF* strain with ObgE^N258^ (ObgE^VU^) (51). The strains were otherwise isogenic, suggesting that one strain was a derivative of 17978VU or 17978UN. Second, LptD is a β-barrel OM protein responsible for the translocation of LPS/LOS in Gram-negative bacteria (58). Third, we previously reported a potential role for undecaprenyl pyrophosphate, synthesized by UppS, in promoting *A. baumannii* envelope integrity in 17978UN ∆*mlaF* (21). The 17978UN allele for each candidate gene (encoding ObgE I258; LptD V799; UppS T78) was substituted in the endogenous locus (encoding ObgE N258; LptD F799; UppS M78) in 17978VU wild-type and 17978VU ∆*mlaF* strains. We reasoned that if the candidate gene contributes to the contrasting phenotypes, then the 17978VU ∆*mlaF* mutant containing the 17978UN candidate allele would exhibit the 17978UN ∆*mlaF* phenotype of increased membrane stress sensitivity. As expected, none of the strains displayed a growth defect when grown in LB alone (Fig. 2C through E). 17978VU ∆*mlaF uppS*^UN^ was unable to grow in SDS/EDTA similar to 17978UN ∆*mlaF*, suggesting that the *uppS* allele determines membrane stress sensitivities of 17978VU and 17978UN ∆*mlaF* strains (Fig. 2H). By contrast, neither the *obgE* nor *lptD* 17978UN alleles altered membrane stress sensitivity in the 17978VU ∆*mlaF* strain (Fig. 2I and J). We observed similar results in an independent dilution spotting experiment (Fig. S1A and B). To test allele prevalence in the ATCC culture stock, we screened 46 isolates from the ATCC 17978 culture received in 2021 for the *uppS* allele and found that one was indeterminant, 43/45 contained *uppS*^UN^ and 2/45 contained *uppS*^VU^(Fig. S1C). Together, these data show that the Mla system is synthetic with *uppS* alleles in *A. baumannii* for resistance to SDS/EDTA membrane stress.

### UppS^UN^ has decreased enzymatic activity, leading to lower cellular Und-P levels

**Fig 3:**
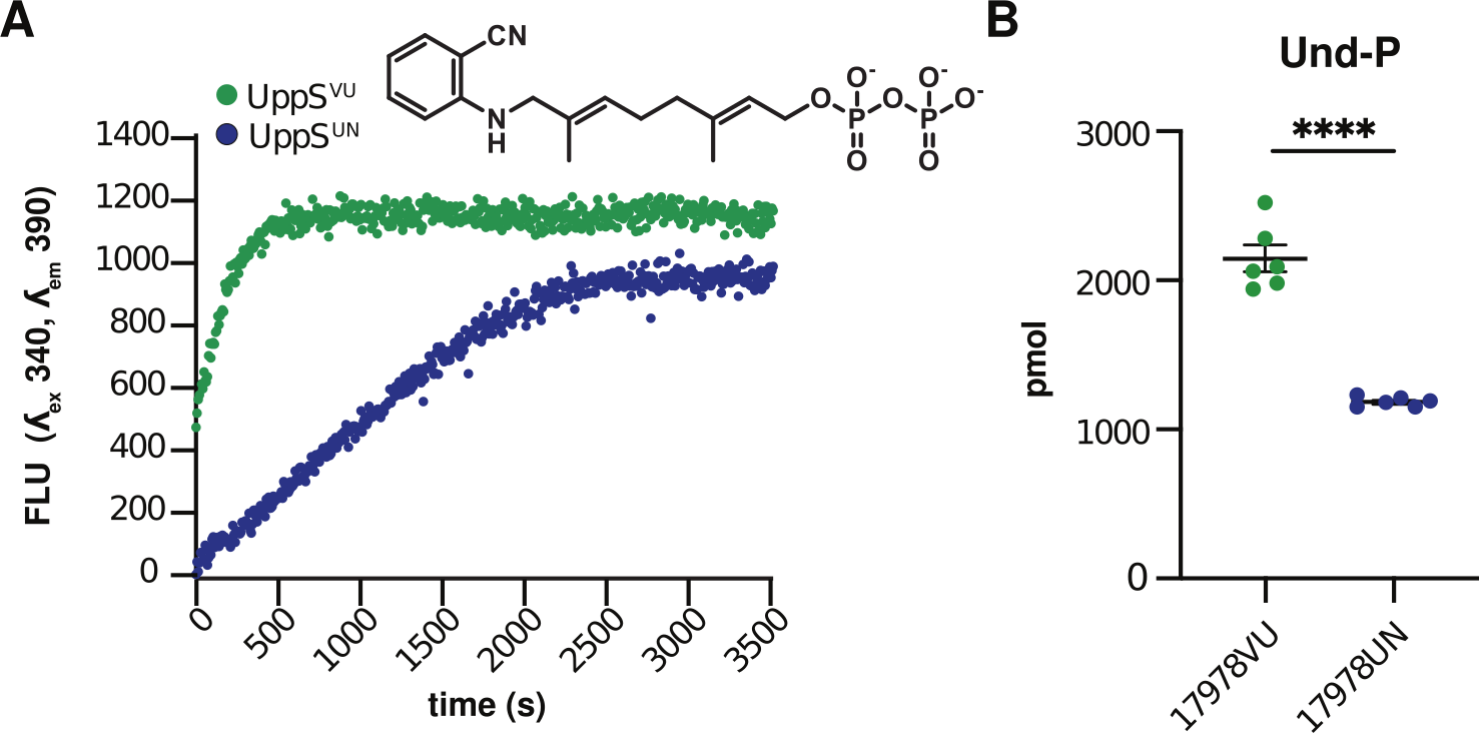
UppS^UN^ demonstrates decreased enzymatic rate and results in decreased cellular Und-P compared to UppS^VU^. (**A**) UppS activity with 2CNA-GPP, a fluorescent analog of the endogenous substrate farnesyl pyrophosphate (FPP). Activity of purified UppS from strains *A. baumannii* ATCC 17978VU (green) and 17978UN (blue) was measured with the 2CNA-GPP substrate analogue and monitoring fluorescence increase upon elongation at 340 nm excitation and 390 nm emission. Data are *n* = 1. Experiments were conducted three independent times with similar results. (**B**) Mass spectrometry quantitation of bacterial C55 undecaprenyl phosphate (Und-P) in ATCC 17978VU and 17978UN wild-type strains. Significance is by unpaired *t*-test. ****P < 0.0001. Data are mean ± SEM, *n* = 6.

Based on results thus far, we hypothesized that UppS^UN^ (T78) was defective compared to UppS^VU^ (M78). First, protein secondary structure was compared by circular dichroism analysis which showed that there were no structural differences between the UppS variants (Fig. S2A and B). Next, enzymatic activity was compared using purified protein and a fluorescent analog (2-nitrileanilinogeranyl diphosphate; 2CNA-GPP) of the UppS substrate farnesyl-pyrophosphate (FPP). UppS from *E. coli, Vibrio vulnificus, Staphylococcus aureus,* and *Bacteroides fragilis* have previously been shown to catalyze the extension of the fluorescent substrate analog 2CNA-GPP (59, 60). Upon elongation of the analog, a concomitant increase in fluorescence can be readily monitored via a microplate assay. By this assay, UppS^UN^ was found to have a fivefold decrease in the enzymatic rate compared to UppS^VU^ (Fig. 3A). To determine if the decreased enzymatic rate of UppS^UN^ results in decreased levels of the essential glycan carrier Und-P in *A. baumannii*, cellular pools of Und-P in the 17978VU and 17978UN wild-type strains were quantified. The 17978UN strain contained twofold lower levels of Und-P compared to the 17978VU wild-type strain (Fig. 3B). Transcript abundance of *uppS* was assessed via reverse transcription quantitative PCR (RT-qPCR) and revealed no differences in abundance between 17978VU and 17978UN (Fig. S2C). Therefore, these data suggest that the UppS^UN^ enzyme has decreased enzyme function that results in decreased cellular levels of Und-P.

### The *uppS*^UN^ allele increases envelope permeability in *∆mlaF* strains

**Fig 4:**
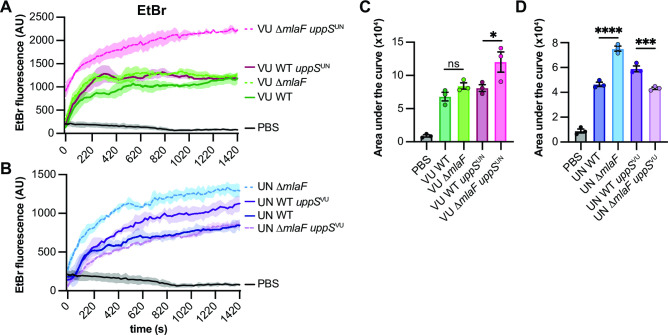
UppS^UN^ increases membrane permeability in Δ*mlaF* mutants. (**A and B**) *A. baumannii* ATCC 17978VU (**A**) and 17978UN (**B**) wild-type and Δ*mlaF* strains with the endogenous or alternate *uppS* allele were incubated with efflux pump inhibitor CCCP and ethidium bromide and fluorescence was measured. (**C and D**) Quantified area under the curve for A and B, respectively. Significance is by one-way ANOVA with Tukey’s multiple comparisons test. All strains were significantly higher than the PBS control (*P* < 0.001). Comparisons between Δ*mlaF* strains with their respective wild-type strain are shown. Significant differences are indicated by *P < 0.05, ***P < 0.001, ****P < 0.0001. Data are means ± SEM, *n* = 3. Experiments were conducted two independent times with similar results.

Given the role of UppS and the Mla system in cell envelope biosynthesis and integrity, we hypothesized that the *uppS*^UN^ allele would increase envelope permeability in ∆*mlaF* mutants. Likewise, we predicted that the *uppS*^VU^ allele would reduce envelope permeability in a 17978UN Δ*mlaF* background. Therefore, the *uppS*^VU^ allele was introduced into 17978UN wild-type and 17978UN ∆*mlaF* strains. An ethidium bromide (EtBr) uptake assay was used to test the effect of the *uppS* alleles on envelope permeability in wild-type and ∆*mlaF* strains (24, 61). In the 17978VU background, ∆*mlaF uppS*^UN^ had significantly increased EtBr permeability compared to wild-type, ∆*mlaF,* and wild-type *uppS*^UN^ (Fig. 4A and C). Similarly, in the 17978UN background, introducing the *uppS*^VU^ reduced EtBr permeability in a ∆*mlaF* mutant (Fig. 4B and D). Together, these data suggest that *uppS* and the Mla system synergize to promote cell envelope integrity.

### Isoprenoid pathway mutations suppress ∆*mlaF* membrane stress sensitivity only in the presence of *uppS*^UN^

**Fig 5:**
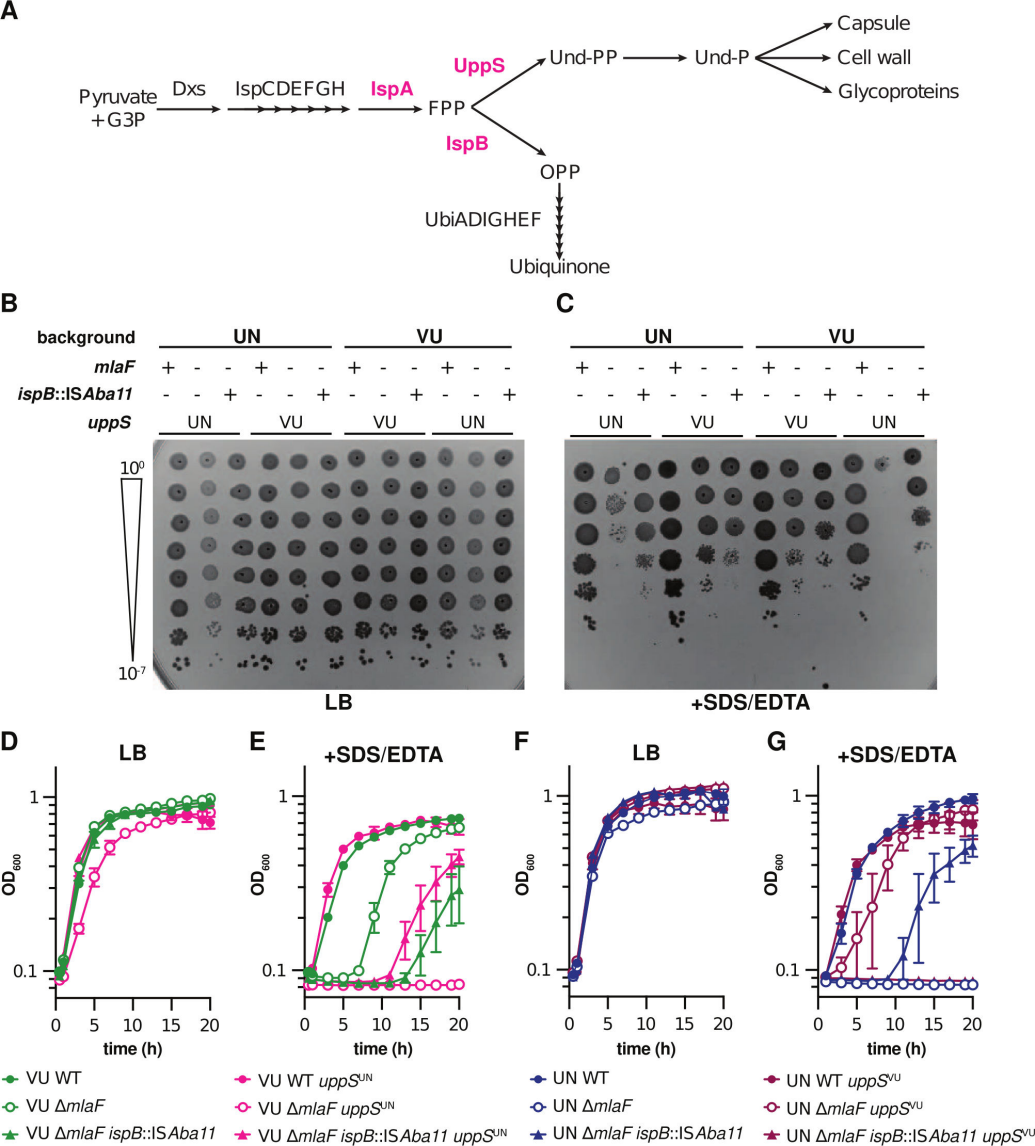
Isoprenoid biosynthesis suppressor mutations only confer increased resistance to SDS/EDTA in *A. baumannii* ATCC 17978 Δ*mlaF* strains with UppS^UN^. (**A**) Schematic of the isoprenoid biosynthetic pathway with key genes highlighted in red. (**B and C**) Wild-type and mutant strains were serially diluted before spotting on LB plates without (**B**) and with (**C**) 0.01% SDS and 0.15 mM EDTA. Experiments were conducted four independent times (*n* = 8) with similar results. (D–G) 17978VU (**D and E**) and 17978UN (**F and G**) wild-type and mutant strains were grown in LB without (**D and F**) or with (**E and G**) 0.01% SDS and 0.175 mM EDTA. Data are means ± SEM, *n* = 3.

We previously identified a suppressor mutation that restored virulence, membrane stress, and antibiotic resistance to wild-type levels in a 17978UN ∆*mlaF* background by decreasing transcript abundance of *ispB* (21). This 17978UN ∆*mlaF ispB*::IS*Aba11* suppressor was included to test the contribution of the isoprenoid biosynthesis pathway to the contrasting phenotypes (outlined in Fig. 5A). Thus, to determine if the isoprenoid biosynthetic pathway impacting Δ*mlaF* phenotypes depends on the *uppS* allele present, 17978UN ∆*mlaF ispB::*IS*Aba11* and 17978VU ∆*mlaF ispB::*IS*Aba11* strains were constructed with the endogenous or alternate *uppS* allele. Dilution spotting to LB agar plates with and without SDS/EDTA determined colony formation efficiency in the presence of membrane stress. As expected, none of the strains exhibited defects in colony formation on LB alone (Fig. 5B). Notably, the 17978UN ∆*mlaF* mutant displayed decreased opacity that was reversed by the presence of *ispB::*IS*Aba11*, as previously reported (21), or *uppS*^VU^. In the presence of SDS/EDTA membrane stress, ∆*mlaF* strains encoding UppS^UN^ exhibited a clear defect in colony formation efficiency compared to ∆*mlaF* strains encoding UppS^VU^ (Fig. 5C). This further supports the conclusion that *uppS* is the genetic cause behind the differences in membrane stress resistance between Δ*mlaF* strains in ATCC 17978VU and 17978UN. Similarly, the *ispB* suppressor restored colony formation on SDS/EDTA in Δ*mlaF* strains encoding the *uppS*^UN^ allele, regardless of strain background (Fig. 5C). This suggests that the *ispB* suppressor function is associated specifically with *uppS*^UN^. We noted that the 17978UN ∆*mlaF* strain often had increased colony formation on SDS/EDTA plates than the 17978VU ∆*mlaF uppS*^UN^ strain which was unexpected; we isolated several colonies of 17978UN ∆*mlaF* from the SDS/EDTA plate and determined that 1/8 appeared to be a stable suppressor but it did not encode the *ispB::*IS*Aba11* allele and the strain was not further investigated. This suggests that *uppS*^UN^ defects may be partially compensated by additional alleles in the 17978UN background.

To examine the effect of the *uppS* alleles on Δ*mlaF* growth over time, we performed growth curves in LB with or without SDS/EDTA. Again, none of the strains showed overt growth defects when grown in LB alone (Fig. 5D and F). However, in the Δ*mlaF* background, the strain with the *uppS*^UN^ allele was unable to grow in media containing SDS/EDTA. Conversely, the *uppS*^VU^ allele conferred partial resistance to SDS/EDTA in the Δ*mlaF* background (Fig. 5E and G). Interestingly, 17978UN Δ*mlaF ispB::*IS*Aba11 uppS*^VU^ was unable to grow in the presence of membrane stressors SDS/EDTA (Fig. 5G). This result suggests that the *uppS*^VU^ allele in a 17978UN ∆*mlaF* background may be hindered by the *ispB* suppressor, perhaps due to a decreased flux toward ubiquinone production.

While constructing the 17978VU ∆*mlaF uppS*^UN^ strain, one isolate displayed a distinct phenotype resembling that of 17978VU ∆*mlaF*. Upon whole-genome sequencing, a mutation was discovered in *ispA* that resulted in a G223E substitution. This mutation was then reconstructed in the 17978UN ∆*mlaF* background to assess its function as a suppressor of the ∆*mlaF uppS*^UN^ phenotype. A dilution spotting assay was used to characterize both *ispA* and *ispB* suppressors in the 17978UN background. All four strains grew similarly on LB medium (Fig. S3). 17978UN ∆*mlaF* displayed a strong growth defect that was partially rescued by suppressor mutations in *ispA* and *ispB* (Fig. S3). These data support a model in which the isoprenoid biosynthetic pathway is synthetic with the Mla system in *A. baumannii*. We predict that the *ispA* and *ispB* ∆*mlaF* suppressors function by increasing the metabolic flux toward UppS and the production of Und-P, which promotes ∆*mlaF* mutant growth in the presence of the *uppS*^UN^ allele.

### UppS^UN^ results in reduced capsule and LOS abundance in the ∆*mlaF* background

**Fig 6:**
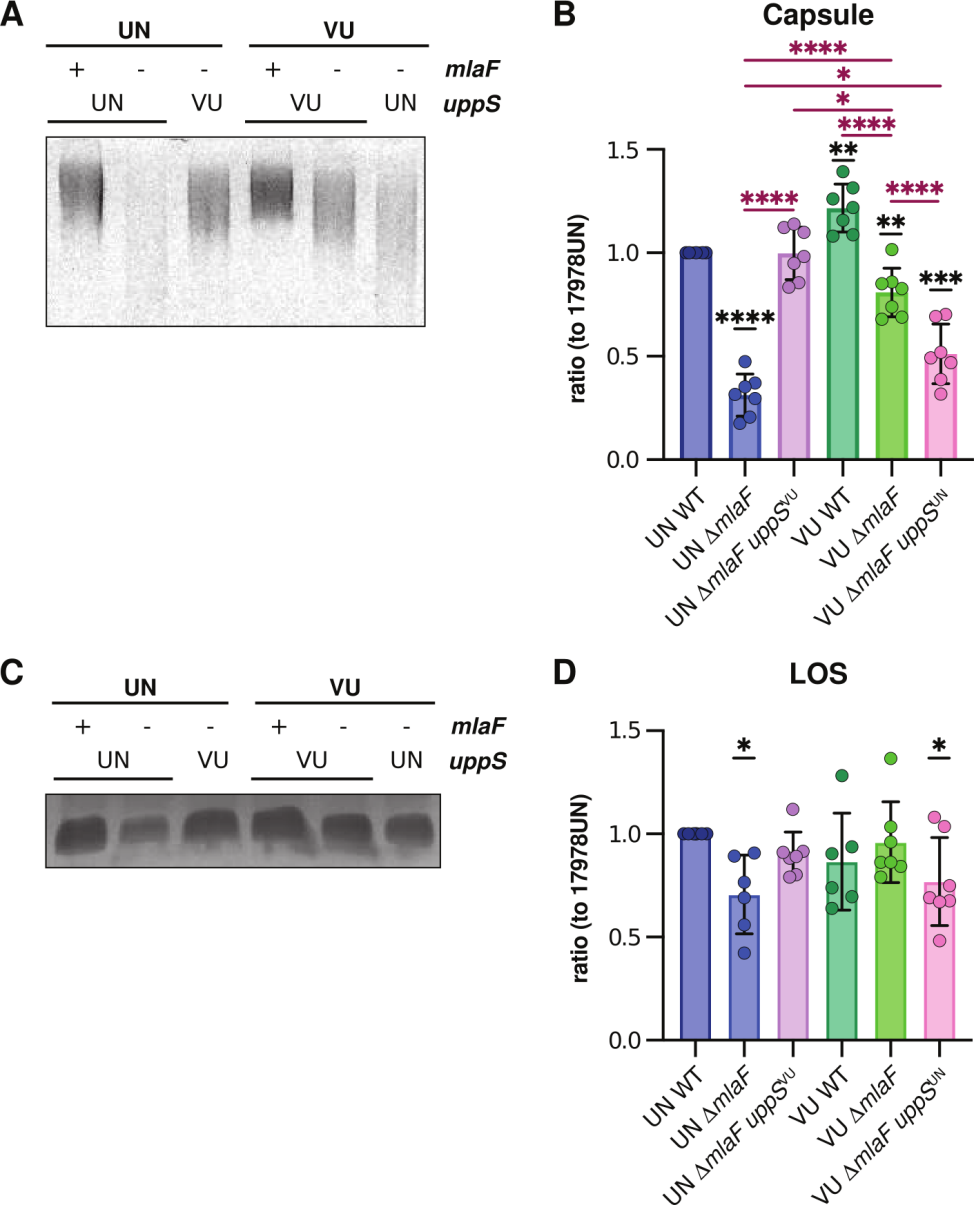
UppS^UN^ results in reduced capsule and LOS abundance. (**A**) Alcian blue stain of capsular polysaccharides from cell lysates. Cell lysates were normalized to total protein. Image is representative of seven biological replicates from three independent experiments. (**B**) Densitometric quantification of capsule bands as a ratio to 17978UN wild type. Data are means ± SEM, *n* = 7 from three independent experiments, significance is by one-sample *t*-test compared to 1 (black asterisk) and one-way ANOVA with Sidak’s multiple comparisons (maroon asterisk). *P < 0.05, **P < 0.01, ***P < 0.001, ****P < 0.0001. (**C**) Silver stain of proteinase K-treated cell lysates to stain for LOS. Cell lysates were normalized to total protein. Image is representative of seven biological replicates from three independent experiments. (**D**) Densitometric quantification of LOS bands as a ratio (to 17978UN wild type). Data are means ± SEM, *n* = 6–7 from three independent experiments, significance is by one-sample *t*-test compared to 1. *P < 0.05.

Und-P has an established role in capsule, peptidoglycan, and glycoprotein biosynthesis; therefore, we tested whether cell wall or capsule is altered by the reduced activity of UppS^UN^. Peptidoglycan staining by 3-[(7-nitro-2,1,3-benzoxadiazol-4-yl)amino]-d-alanine hydrochloride (NADA) staining showed no dramatic differences among strains (Fig. S4A). However, capsule staining by Maneval suggested there may be subtle differences in the capsule among 17978UN and 17978VU Δ*mlaF* strains (Fig. S4B). Therefore, capsule polysaccharide was visualized and quantified by Alcian blue staining. Alcian blue revealed that Δ*mlaF* strains with *uppS*^UN^ alleles had dramatically different polysaccharide distributions which were more diffuse and consistent with decreased molecular weight (Fig. 6A; Fig. S4C). Gel densitometry quantification showed that Δ*mlaF* strains with *uppS*^UN^ alleles also had decreased capsule abundance compared to 17978UN wild type (Fig. 6B). This analysis also showed that 17978VU wild type had increased capsule compared to 17978UN (Fig. 6A and B). Overall, Δ*mlaF* strains expressing *uppS*^UN^ had further reduced capsule when compared to the 17978UN wild type than the same strain background expressing *uppS*^VU^ (Fig. 6A and B); however, the *uppS* allele did not fully restore these differences, suggesting other genetic differences between 17978UN and 17978VU also contribute to capsule abundance.

Next, we tested whether the UppS^UN^ variant was required for the decreased LOS abundance in the 17978UN Δ*mlaF* mutant we previously reported (21). Although Und-P has an established role in the production of LPS as the lipid carrier of O-antigen precursors in other Gram-negative organisms (15), there is no known role for Und-P in the biosynthesis of LOS in *A. baumannii*. We hypothesized that reduced LOS abundance in the 17978UN ∆*mlaF* mutant would depend on the presence of the *uppS*^UN^ allele. Indeed, when LOS was quantified by silver staining of proteinase K-treated cell lysates, Δ*mlaF* strains with *uppS*^UN^ had reduced LOS abundance (Fig. 6C and D; Fig. S4D). The wild-type strains and the Δ*mlaF* strains with *uppS*^VU^ showed no reduction in LOS (Fig. 6C and D; Fig. S4D). Together, these results suggest that UppS activity and the Mla system are important for maintaining capsule and LOS abundance in *A. baumannii*.

### Mla and UppS synergy influences clinically relevant phenotypes

**Fig 7:**
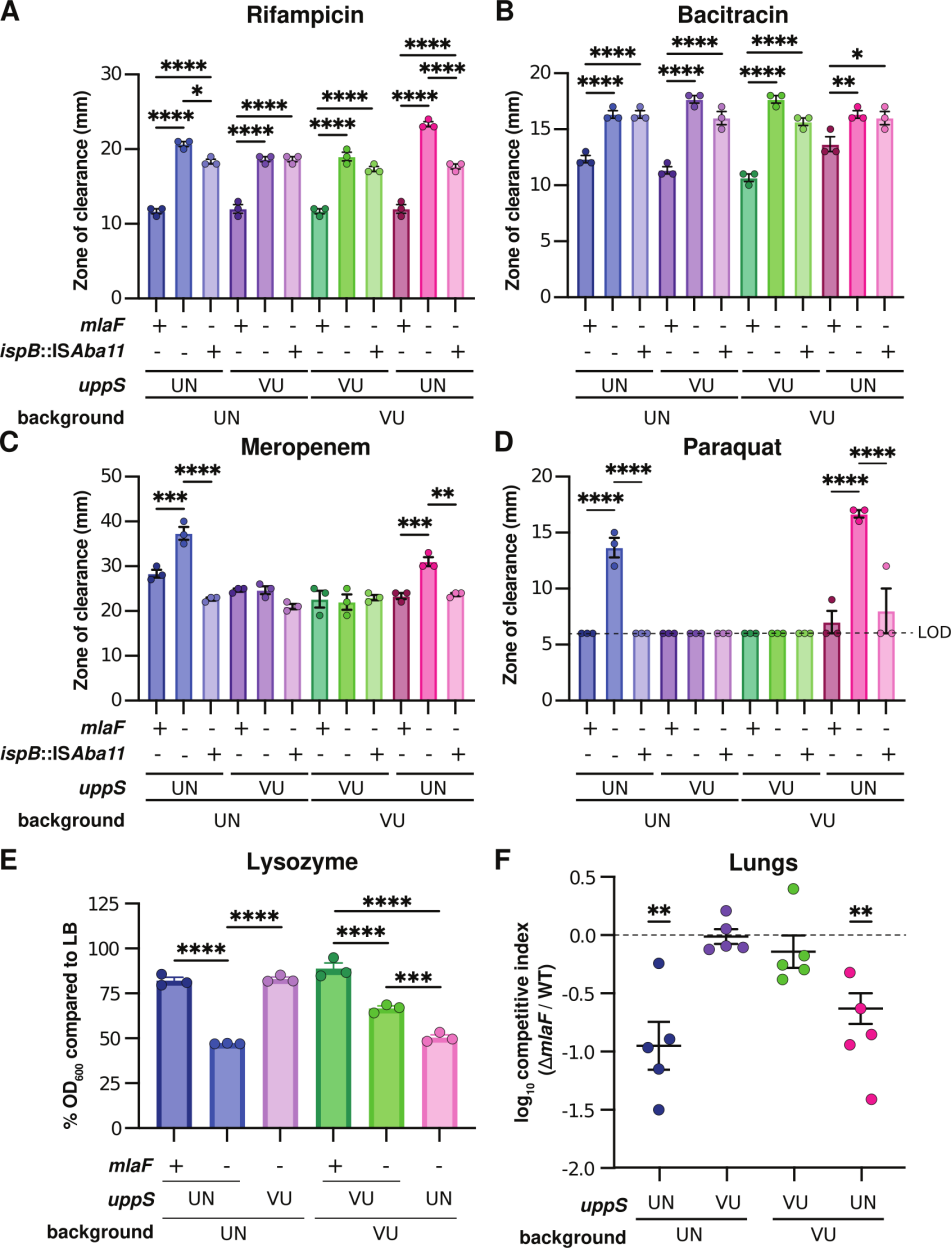
In an Δ*mlaF* background, UppS^UN^ decreases antimicrobial resistance and virulence in a murine model of pneumonia. (A–D) Antimicrobial susceptibility of *A. baumannii* ATCC 17978VU and 17978UN wild-type and mutant strains was determined by a disk diffusion assay and measuring the zone of clearance. Experiments were conducted three independent times with similar results. Data are mean ± SEM, *n* = 3. Significance is by one-way ANOVA with Tukey’s multiple comparisons. Limit of detection (LOD) = 6 mm. (**E**) Lysozyme susceptibility of 17978VU and 17978UN wild-type and mutant strains at 12 h during growth with a final concentration of 1 mg/mL lysozyme. Data are mean ± SEM, *n* = 3. Significance is by one-way ANOVA with Tukey’s multiple comparisons. (**F**) 17978VU and 17978UN wild-type and Δ*mlaF* strains with the endogenous or alternate *uppS* allele were used to intranasally infect C57BL/6 mice in a 1:1 wild-type:mutant ratio. After 48 h post infection, the lungs were harvested and bacterial burdens were enumerated. Data are mean ± SEM, *n* = 5. Normality of log_10_-transformed competitive index data was determined by the Kolmogorov-Smirnov test, and significance was determined by a one-sample *t*-test compared to 0. *P < 0.05, **P < 0.01, ***P < 0.001, ****P < 0.0001.

Previous reports showed that *A. baumannii mla* mutants have increased susceptibility to antimicrobials such as gentamicin, novobiocin, rifampicin, meropenem, and the superoxide donor paraquat (21, 24, 51). We hypothesized that in a ∆*mlaF* mutant background, the presence of the *uppS*^UN^ allele would result in increased susceptibility to antimicrobials compared to strains with the *uppS*^VU^ allele. In a disk diffusion assay, the presence of *uppS*^UN^ in a ∆*mlaF* mutant resulted in increased susceptibility to antimicrobials from multiple classes regardless of the ATCC 17978 background, represented by increased zone of clearance diameters (Fig. 7A through D; Fig. S5A and B). These antimicrobials included first line antibiotics such as meropenem and imipenem. These results show that UppS synergizes with the Mla system to promote *A. baumannii* antimicrobial resistance. Consistent with above findings, the *ispB* suppressor primarily restored resistance to strains containing the *uppS*^UN^ allele. We further hypothesized that the *uppS*^UN^ allele would confer decreased resistance of ∆*mlaF* strains to lysozyme, a host antimicrobial enzyme that cleaves the peptidoglycan cell wall (62). During growth in LB with 1 mg/mL lysozyme, the 17978UN ∆*mlaF* exhibited severely reduced resistance to lysozyme compared to the 17978UN wild-type strain (Fig. 7E; Fig. S5A). Resistance was restored to 17978UN ∆*mlaF* by the *uppS*^VU^ allele. Similarly, the *uppS*^UN^ allele conferred decreased lysozyme resistance to 17978VU ∆*mlaF* (Fig. 7E; Fig. S5A). Together, these data show that the *uppS*^UN^ allele decreases resistance to multiple antimicrobial stresses in a ∆*mlaF* mutant.

We previously reported that the ∆*mlaF* mutant in the ATCC 17978UN background had a virulence defect in a murine model of pneumonia (21). Based on decreased resistance to host stresses, we hypothesized that the ∆*mlaF uppS*^UN^ strain would have a greater virulence defect than the ∆*mlaF uppS*^VU^ strain. Therefore, we compared bacterial burdens in a competitive infection with wild-type 17978VU and 17978UN strains and ∆*mlaF* mutants where the mutant expressed either the endogenous or alternate *uppS* allele. At 40 h post infection, the ∆*mlaF* mutants containing the *uppS*^UN^ allele had a significant defect in virulence compared to wild-type regardless of strain background (Fig. 7F; Fig. S5B through E). The ∆*mlaF* mutant strains with *uppS*^VU^ showed no virulence defect compared to wild type (Fig. 7F; Fig. S5B through E). This demonstrates that the virulence defect observed in ∆*mlaF* is due to synergy with the *uppS*^UN^ allele. Together, these data suggest that the Mla system and Und-P levels synergize to promote antimicrobial resistance and virulence in *A. baumannii*.

## DISCUSSION

The *A. baumannii* cell envelope is the first line of defense against antibiotic stress and the host. Therefore, it is critical to understand the biological processes that uphold this barrier. The Mla system is an important factor in maintaining outer membrane lipid asymmetry to promote envelope integrity in Gram-negative bacteria. Multiple reports have demonstrated envelope defects in the absence of the Mla system in *A. baumannii* (16, 24, 51). Here, we found that the deletion of *mlaF* in two closely related *A. baumannii* ATCC 17978 strains results in different phenotypes. Genetic dissection uncovered synergy between Und-P abundance and the Mla system in *A. baumannii*.

*A. baumannii* ATCC 17978 is a commonly used type strain. We recently discovered that ATCC 17978 was a mixed culture of two closely related strains that differed by the accessory locus AbaAL44 and multiple SNPs (54). Depending on the time of order, the ratio of 17978UN to 17978VU isolates received from ATCC varied. In 2009, 4/6 isolates screened were 17978UN and 2/6 were 17978VU; by contrast, in an ATCC order from 2021, 35/36 were 17978UN and 1/36 were 17978VU (54). This suggests that while the 17978UN allele of *uppS* is uncommon in circulating *A. baumannii* strains (Fig. 1), the majority of recent 17978 isolates from ATCC are likely 17978UN. Differences in ∆*mlaF* phenotypes of ATCC 17978UN and 17978VU presented the opportunity to use these variants as a genetic tool to investigate synthetic gene pairs. Ultimately, *uppS* was shown to synergize with ∆*mlaF* in resistance to membrane stresses, envelope permeability, antibiotic and host stress resistance, and virulence in a mouse lung infection model. This result was surprising as a previous report identified SNPs in *obgE,* which encodes an essential GTPase involved in the stringent response, as the critical determinant of differences in growth and the stringent response in two isogenic ATCC 17978 variants (51). While we did not observe overt differences in growth based on the *obgE* allele, we speculate that is likely due to differences in growth conditions such as aeration. Interestingly, among the protein-encoding genetic differences between 17978VU and 17978UN, the 44 kb AbaAL44 locus and *lptD*^UN^ are the only 17978UN alleles that are more common than 17978VU alleles (Fig. 1). This may suggest that there may have been selective pressure to maintain *lptD*^UN^ in the 17978UN background. Together, these findings exemplify that closely related strains can be leveraged to uncover underlying integration of essential biological process.

As an essential glycan carrier, Und-P plays a role in the biosynthesis of multiple bacterial cell envelope components including peptidoglycan, glycoproteins, LPS O-antigen, lipid A, and capsular polysaccharides (53, 63, 64). However, *A. baumannii* does not synthesize the LPS O-antigen and does not encode LpxT that uses Und-PP as a phosphate donor for lipid A. Imaging of isogenic strains with UppS variants showed no overt difference in peptidoglycan (Fig. S3A). Staining for capsular polysaccharide from whole cell lysates revealed reduced capsule in Δ*mlaF* mutants and further reduced capsule in Δ*mlaF* mutants expressing *uppS*^UN^. Differences between Δ*mlaF* strains expressing the same *uppS* allele but in opposing 17978 backgrounds suggest that there may be additional synergistic relationships impacting capsule content. UppS^UN^ has a decreased enzymatic rate and confers increased membrane sensitivity in an Δ*mlaF* background. However, there were no defects in the wild-type strains for membrane stress susceptibility, suggesting that the Δ*mlaF* defects synergize with reduced Und-P to result in a weakened cell envelope. We previously observed that *A. baumannii* ATCC 17978UN ∆*mlaF* had decreased LOS abundance (21), suggesting the Mla system is important for LOS abundance in *A. baumannii*. Here, we show that the reduction in LOS within 17978UN ∆*mlaF* is due to UppS^UN^, as an isogenic mutant with UppS^VU^ displays wild-type-like LOS levels. This suggests that Und-P may have an uncharacterized role in LOS synthesis in *A. baumannii*. For example, Und-P could serve as a glycan carrier for LOS such as for the sugar molecules in the core oligosaccharides or a non-homologous enzyme may use Und-PP as a phosphate donor for lipid A similar to LpxT. In many gammaproteobacteria, the *uppS* gene is in an operon with a phosphatidate cytidylyltransferase, an enzyme important in phospholipid biosynthesis, suggesting there may be a broader synergistic relationship between the transport and biosynthesis of phospholipids and other envelope components such as capsule, peptidoglycan, and glycoproteins. In subpopulations of *A. baumannii* that have been evolved to be LOS deficient, the Mla system hinders fitness (39). However, multiple *A. baumannii* strains with LOS deficiency showed upregulation of Mla genes (12, 65). This demonstrates a distinction between populations that have adapted to LOS deficiency and non-adapted populations with LOS deficiency. In our work, UppS^UN^ reduces but does not fully deplete LOS in the ∆*mlaF* strain background. We, therefore, show that the Mla system is important for maintaining cell envelope integrity in the presence of reduced Und-P.

Suppressor mutants in genes encoding isoprenoid biosynthetic pathway enzymes restored membrane stress resistance to Δ*mlaF uppS*^UN^ strains. We found that the Δ*mlaF uppS*^UN^ phenotype was suppressed by mutations in isoprenoid biosynthetic genes *ispA* and *ispB*. The IS*Aba11* transposition to the 5′ untranslated region of *ispB* was also found in an extensively drug resistant clinical isolate of *A. baumannii*, demonstrating potential clinical implications for this insertion (66). Here, we identified another suppressor in an isoprenoid biosynthetic gene encoding IspA^G223E^ that arose when constructing the 17978VU Δ*mlaF uppS*^UN^ strain. In *E. coli*, *ispA* null mutants had reduced isoprenoid quinone levels that was rescued by the overexpression of either *ispB* or *ispU* (*uppS*) (67, 68). This suggests that there may be low-level functional redundancy between IspA, IspB, and UppS. We, therefore, hypothesize that IspA^G223E^ enhances low-level Und-PP synthesis performed by IspA. Similarly, in *E. coli*, a defective UppS variant resulted in growth defects that were suppressed by mutations in isoprenoid pathway genes (69). This suggests that there may be a conserved mechanism to promote the production of undecaprenyl species in the presence of a defective UppS.

The structure of UppS has been solved from multiple organisms, including *A. baumannii* (70–72). The substitution in UppS that differs between 17978VU (M78) and 17978UN (T78) is at the 78th amino acid position, located on the α3 helix. The α3 helix borders the active site and surrounds the open cavity the product chain would occupy. Residues along the α3 helix are largely conserved (73). The methionine at this position on the α3 helix is conserved in prenyl-transferases from *E. coli*, *Saccharomyces cerevisiae*, *Mycobacterium tuberculosis*, *Arabidopsis thaliana*, and humans (74). This suggests that the methionine is important for the structure or function of prenyl-transferases. In the 17978UN UppS variant, the methionine at position 78 is replaced with a threonine. While not highlighted as a critical residue, the surrounding hydrophobic residues near *E. coli* UppS M86 (M78 in *A. baumannii*) are implicated in interactions with the substrate (74). Importantly, there were no major structural differences in UppS between the M78 and T78 variants by circular dichroism analysis (Fig. S1A and B). The substitution to a threonine in 17978UN may, therefore, perturb internal hydrophobic interactions between the α3 helix and substrate. We found that 17978UN UppS has a reduced enzymatic rate compared to 17978VU UppS. This suggests that the T78 encoded by ATCC 17978UN may perturb crucial interactions with the substrate hydrophobic tail required for efficient Und-PP production.

Isoprenoid biosynthesis is essential for diverse processes in bacteria ranging from metabolism to virulence at the host-pathogen interface. As such, proteins within isoprenoid biosynthesis have been considered a drug target for novel therapeutics (75, 76). Furthermore, UppS specifically is a proposed drug target for *A. baumannii* (77). The synthetically sensitive Δ*mlaF* mutants in ATCC 17978UN suggest that targeting UppS or depleting undecaprenyl species in a combination therapy may represent an effective therapeutic strategy. The cell envelope is the largest barrier to effective antibiotic treatment in Gram-negative bacteria. The epistatic interaction between the *A. baumannii* Mla system and UppS identifies a potential strategy to circumvent the envelope barrier for difficult-to-treat infections. Our findings provide a deeper understanding into how *A. baumannii* maintains cell envelope integrity to survive in hostile environments.

## MATERIALS AND METHODS

### Bacterial strains and growth

All bacterial strains and plasmids used in this study are listed in Tables S1 and S2, respectively. Strains were grown in LB or on LB plates with 1.5% (wt/vol) agar. Antibiotics were used at the following concentrations: carbenicillin, 75 mg/L; kanamycin, 40 mg/L; chloramphenicol, 15 mg/L. Overnight cultures were started in 3–5 mL LB, inoculated with a single colony, and incubated at 37°C while shaking at 180 rpm for 8–16 h. Growth curves were conducted in 100 µL media in a flat bottom 96-well plate, inoculated with 1 µL overnight culture, and incubated at 37°C with shaking. Bacterial growth was monitored by optical density at 600 nm (OD_600_) in an EPOCH2 BioTek (Winooski, VT) plate reader. For assays on membrane stress, SDS/EDTA was included in the media at varying concentrations and the concentrations used are noted in each figure due to variability. For lysozyme susceptibility assays, lysozyme was included in the media at a final concentration of 1 mg/mL and the OD_600_ at 12 h was used to calculate the %OD_600_ compared to the average of the OD_600_ at 12 h in LB for each strain.

### Plasmid construction and allelic exchange mutants

All oligonucleotides used are listed in Table S3. DNA was amplified using 2× Phusion Master Mix (ThermoFisher, Waltham, MA), Q5 High Fidelity 2× Master Mix [New England Biolabs (NEB), Ipswich, MA], or GoTaq Green Master Mix (NEB, Ipswich, MA). The pFLP2 vector was used for all allelic exchange mutants. Using ATCC 17978VU or 17978UN as the template, 1,000 bp upstream and downstream of the mutation of interest was amplified. For pFLP2-*obgE*, HN1 and HN2 were used. For pFLP2-*lptD*, HN23 and HN24 were used. For pFLP2-*uppS*, HN25 and HN26 were used. For ispA* reconstruction, HN85 and HN86 were used with strain LP546 as the template. The PCR product was incorporated into a digested pFLP2 backbone using KpnI and BamHI restriction sites and HiFi ligation mix (NEB, Ipswich, MA). All restriction enzymes are from NEB (Ipswich, MA). Strains containing the *ispB* suppressor mutation and *mlaF* knockout were generated using pLDP70 and pLDP8, respectively, (21). *A. baumannii* was transformed through conjugation by triparental mating with *E. coli* strain HB101 containing pRK2013 as the helper strain. Merodiploids containing the integrated pFLP2 vector were screened on plates containing 10% sucrose and 75 mg/L carbenicillin and Suc^S^ and Carb^R^ colonies were selected. Merodiploids with the appropriate phenotype were screened by PCR to confirm plasmid incorporation at the correct site. Merodiploids were grown on LB agar, resuspended in LB, and plated to LB agar containing 10% sucrose to select for second crossover events, and the Suc^R^ strains were screened for Carb^S^. Genotypes were confirmed via Sanger Sequencing (UIC Genomics Research Core) and/or whole-genome sequencing (SeqCoast Genomics, Portsmouth New Hampshire; SeqCenter, Pittsburg PA) to confirm no additional mutations in relevant loci.

UppS expression plasmids for protein purification were generated using pET-15b digested with BamHI and NdeI. UppS was amplified from either 17978VU or 17978UN with HN94 and HN95 and ligated into the pET-15b backbone by HiFi ligation. All plasmid sequences were confirmed with Sanger sequencing by the UIC Genomics Research Core or whole plasmid sequencing by Primordium (Monrovia, CA).

To distinguish the *uppS*^UN^ allele from the *uppS*^VU^ allele, primers HN31 (UN) and HN32 (VU) were used with HN30, 2× Green Gotaq (Promega, Madison, WI), and an annealing temperature of 61.9°C and extension time of 2 min.

### Serial dilution spotting assays

Overnight cultures were 10-fold serially diluted in 96-well plates in 1× PBS to 10^−7^. Dilutions were spotted in 3 or 5 µL drops on LB agar and LB agar containing SDS/EDTA and incubated at 37°C overnight. Plates were imaged with a BioTek (Winooski, VT) ChemiDoc MP imager.

### Antibiotic susceptibility assay

Melted soft agar (0.8% agar, 0.8% NaCl) was brought to 50°C in 9.5 mL aliquots before inoculation with 270 µL overnight culture and plating to a prewarmed 15 cm LB agar plate. Once solidified, pre-loaded antibiotic discs [BD (Becton Dickinson), Franklin Lakes, NJ] were placed using the BD automatic disc dispenser onto the agar overlay, and the plates were incubated at 37°C overnight. The following day, diameters of the zones of clearance were measured in mm.

### Ethidium bromide uptake assay

The EtBr uptake assay was adapted from previous studies (24, 61). Bacteria were grown in 3 mL LB to mid-log phase before centrifuging and normalizing to ~1 × 10^10^ CFU in PBS. Normalized cells were plated to confirm equal CFU across strains. In 200 µL final volume, normalized cells were combined with 200 µM carbonyl cyanide 3-chlorophenylhydrazone (CCCP) and 1.2 µM EtBr. EtBr uptake was monitored in a black 96-well plate with reads every 15 s on a BioTek Synergy H1 (Winooski, VT) using excitation and emission wavelengths of 530 nm and 590 nm, respectively.

### UppS purification

UppS^VU^ and UppS^UN^ were purified from BL21 ArcticExpress DE3 RIL cells (VWR, Radnor, PA). Cells expressing pET-15b-UppS^VU^ or pET-15b-UppS^UN^ were grown in 10 mL LB containing carbenicillin overnight at 37°C. The following day, cells were subcultured into 1 L LB containing carbenicillin in a 2.8-L Fernbach flask and grown to mid-log phase at 37°C. At OD_600_ of 0.6, IPTG was added to a final concentration of 0.5 mM to induce protein expression. Cells were incubated overnight while shaking (180 rpm) at 25°C. The following day cells were centrifuged at 2,000 × *g* for 10 min and the cell pellet was lysed using 8 mL of B-PER Bacterial Protein Extraction Reagent (Thermo Scientific, Waltham, MA) per gram of pellet with gentle shaking for 1 h. The lysate was pelleted by centrifugation at 4,300 × *g* for 5 min. The soluble fraction in the supernatant was mixed with an equal part lysis buffer (50 mM NaH_2_PO_4_, pH 8.0, 300 mM NaCl, 10 mM imidazole) and was added to 8 mL Ni-NTA resin (Qiagen, Hilden, Germany) preequilibrated with lysis buffer and rocked for 1 h at 4°C. Protein-bound resin was then applied to a 10 mL chromatography column (BioRad, Hercules, CA) and washed twice with 10 mL wash buffer (50 mM NaH_2_PO_4_, pH 8.0, 300 mM NaCl, 20 mM imidazole). Protein was eluted using elution buffer (50 mM NaH_2_PO_4_, pH 8.0, 300 mM NaCl, 250 mM imidazole) in serial 2 mL elution volumes. Samples were separated by SDS-PAGE and stained with SimplyBlue Safe Stain (Invitrogen, Waltham, MA) to verify protein purification. Protein was desalted using PD-10 desalting columns (Cytiva, Marlborough, MA) into circular dichroism and UppS assay buffers (see SI and below).

### Undecaprenyl-phosphate quantification

Cultures of 17978UN and 17978VU in 5 mL LB were grown to mid-log phase with a final OD_600_ of 0.5. Cultures were pelleted and stored at −80°C. Pellets were subjected to Bligh and Dyer extraction and dried overnight. The crude cell lysate was then resuspended in 200 µL of *n*-propanol and 0.1% ammonium hydroxide (1:3) (78). After sonicating the cell suspension using water bath, 5 µL of sample was injected into C18 column and analyzed for C55 BP/Und-P *m/z* ratio of 845.7 by liquid chromatography/mass spectrometry (LC-MS). Area under the curve of each Und-P peak of all samples was recorded and used to calculate Und-P (pmol) using a standard curve generated from known Und-P concentrations.

### UppS microplate enzyme assays

2CNA-GPP was prepared as described previously (59, 69). In each well of a black-walled 96 well plate, reactions were prepared with 2.5 mM 2CNA-GPP, 0.5 mM MgCl_2_, 5 mM KCl, 0.1% Triton-X-100, and 100 nM recombinant UppS from 17978UN or 17978VU. The plate was incubated in the plate reader at 30°C for 5 min and then, the reaction was initiated with the addition of 1 mM IPP (final concentration). Fluorescence was monitored at 30°C over 1 h at an excitation wavelength of 340 nm and emission wavelength of 390 nm. The reaction rate was determined based on the linear fluorescence increase over the first 8 min for proteins from both strains (*n* = 3).

### Capsule

Samples were prepared similarly as previously described (79). Briefly, 3-mL overnight cultures were diluted 1:100 in 3 mL LB and grown to early stationary phase with an OD_600_ of 1.4. Two 1-mL aliquots were harvested. One aliquot was boiled at 85°C for 15 min and subjected to a Pierce 660 (Thermo Fisher, Waltham, MA) total protein quantification following manufacturer instructions. The second aliquot was pelleted and resuspended in lysis buffer (60 mM Tris, pH 8, 10 mM MgCl_2_, 50 µM CaCl_2_, 3 mg/mL lysozyme, 60 U/mL DNase, 10 µg/mL RNase) normalized to 5 mg/mL total protein, which was determined to be in the linear range of the capsule densitometry analysis by a dilution series experiment and incubated at 37°C for 1 h. Samples were subjected to three freeze/thaw cycles followed by further DNase and RNase treatment and incubation for 30 min at 37°C. Samples were then treated with SDS to a final concentration of 0.5% and incubated at 37°C for 30 min, followed by 40 μg proteinase K treatment and incubation at 60°C for 1 h. Laemmli sample buffer (Bio-Rad, Hercules, CA) supplemented with DTT to 54 mg/mL was then added before gel electrophoresis on a 4%–12% Bis-Tris gel (MilliporeSigma, Burlington, MA). Fifteen micrograms of total protein was loaded for each sample. Gels were then stained with 0.1% Alcian blue in 40% ethanol/60% 20 mM sodium acetate pH 4.75. Images were captured on a BioTek (Winooski, VT) ChemiDoc MP imager.

### LOS silver stain

Samples were prepared similar to previous descriptions (80, 81). Three milliliters of overnight cultures was diluted 1:100 in 5 mL fresh LB and grown to mid-log with an OD_600_ of 0.5. Two 1mL aliquots were harvested. One aliquot was boiled at 85°C for 15 min and subjected to a Pierce 660 (Thermo Fisher, Waltham, MA) total protein quantification following manufacturer instructions. The second aliquot was pelleted and resuspended in 1× Novex SDS sample buffer (Invitrogen, Waltham, MA) so that the final total protein concentration was 6 mg/mL, which was determined to be in the linear range of the LOS densitometry analysis by a dilution series experiment. Samples were lysed via boiling for 15 min at 85°C and treated with proteinase K at a final concentration of 0.16 µg/µL. Gels were loaded with samples containing 10 µg total protein before electrophoresis on a 16.5% Tris Tricine gel (Bio-Rad, Hercules, CA). Gels were stained with the Pierce Silver Stain kit (Thermo Fisher, Waltham, MA) following manufacturer instructions. Images were captured on a BioTek (Winooski, VT) ChemiDoc MP imager.

### Densitometry analysis of capsule and LOS gels

Densitometry analysis was performed using ImageJ. Images were converted to an 8-bit image, and the background was subtracted using a 50-pixel rolling ball radius. Using the gel analysis tool, bands were selected and peaks were quantified. Data are presented as a ratio to the density of 17978UN wild type for each independent gel.

### Murine model of *A. baumannii* lung infection

Six-week-old female C57BL/6 mice were purchased from Jackson Laboratory. Mice were housed in a temperature-controlled environment with 12 h light/dark cycles and food and water were provided as needed and were acclimated to the facility to 1 week prior to infection. Mice were anesthetized with ketamine/xylazine and inoculated intranasally with 35 µL bacterial suspension 1:1 mixture *A. baumannii* ATCC 17978 wild-type and Δ*mlaF* mutant derivatives, containing approximately 3 × 10^8^ CFU as described previously (21). The inoculum dose was confirmed by serial dilution and plating on selective agar media. Mice were euthanized at 48 h post infection by CO_2_ asphyxiation, and the lungs were excised aseptically. Tissues were homogenized, and all samples were serially diluted and plated on LB and kanamycin selective agar plates for bacterial enumeration. The competitive index of mutant/wild-type strains was calculated by dividing the mutant CFU ratio (CFU_output_/CFU_input_) by the wild-type CFU ratio. All animal care protocols were approved by the University of Illinois Chicago Institutional Animal Care and Use Committee (IACUC; protocol number 20–165) in accordance with the Animal Care Policies of UIC, the Animal Welfare Act, the National Institutes of Health, and the American Veterinary Medical Association (AVMA). Animals were humanely euthanized consistent with the AVMA guidelines.

### Data reporting, statistical analysis, and figure preparation

Each measurement was taken from a distinct biological sample (e.g., bacterial culture from a single colony or an individual mouse). Data processing and statistical analyses were performed using Microsoft Excel 16.77.1 and GraphPad Prism 10. Statistical tests used are indicated in each figure legend. Figures were prepared in Adobe Illustrator 28.1.

## Data Availability

Whole genome sequencing data is available in the National Center for Biotechnology Information (NCBI) sequence read archive (SRA) under BioProject: PRJNA1020123 and PRJNA656143 (22).

## SUPPLEMENTAL MATERIAL

Supplemental material mbio.02804-23-s0001.pdf Figures S1-S5, Tables S1-3, and supplemental text.

Table S4 mbio.02804-23-s0002.xlsx Proteomes and alleles depicted in Figure 1.

ASM does not own the copyrights to Supplemental Material that may be linked to, or accessed through, an article. The authors have granted ASM a non-exclusive, world-wide license to publish the Supplemental Material files. Please contact the corresponding author directly for reuse.
