# Supplementary material for: Genetic synergy between Acinetobacter baumannii undecaprenyl phosphate biosynthesis and the Mla system impacts cell envelope and antimicrobial resistance
Source: mBio. 2024 Feb 16;15(3):e02804-23. doi: 10.1128/mbio.02804-23 (PMC10936186; doi:10.1128/mbio.02804-23)
Supplement: Supplemental material — Figures S1-S5, Tables S1-3, and supplemental text. [file mbio.02804-23-s0001.pdf]

Supplementary information for
Genetic synergy between *Acinetobacter baumannii* undecaprenyl phosphate biosynthesis and the Mla system impacts cell envelope and antimicrobial resistance

Hannah R. Noel, Sowmya Keerthi, Xiaomei Ren, Jonathan D. Winkelman, Jerry M. Troutman, Lauren D. Palmer

Correspondence: ldpalmer@uic.edu

This PDF includes:
Figures S1 to S5
Tables S1 to S3
Supplemental methods
Supplemental references

Figure S1. Dilution spotting of 17978UN and 17978VU wild-type and Δ*mlaF* strains 17978VU strains with *obgE*, *lptD*, and *uppS* alleles from 17978UN, and PCR on the ATCC17978 stock.
(A) Overnight cultures were serially diluted in PBS before plating on an LB agar plate. Image is representative of 5 biological replicates from 2 independent experiments.
(B) Overnight cultures were serially diluted in PBS before plating on an LB agar plate with 0.01% SDS + 0.25 mM EDTA. Image is representative of 5 biological replicates from 2 independent experiments.
(C) Colony PCR on single colonies from ATCC 17978 (2021) stock using primers that distinguish the *uppS* allele between 17978VU and 17978UN. n = 46 with 43 isolates positive for *uppS*^{UN}, 2 isolates positive for *uppS*^{VU}, and one indeterminant.

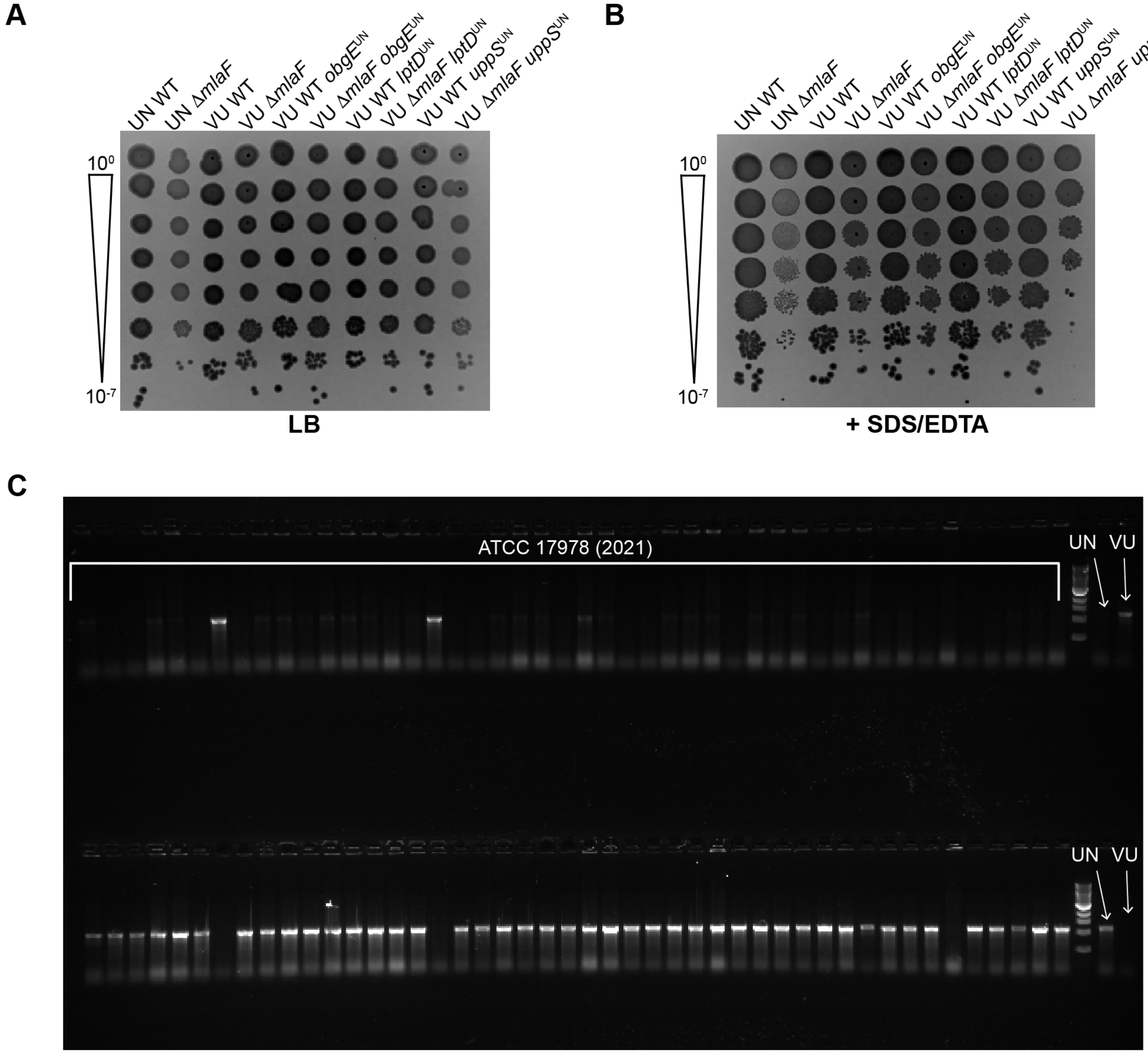

Figure S2. Comparison of UppS^{VU} and UppS^{UN} protein secondary structure and transcript abundance

(A-B) Purified UppS^{VU} and UppS^{UN} were subjected to circular dichroism analysis with a range of 190 nm to 210 nm. N=1.

(C) Transcript abundance of *uppS* in 17978VU wild-type compared to 17978UN wild-type. Data are mean ± SEM, n=3. Not significant by one sample T test.

A

B

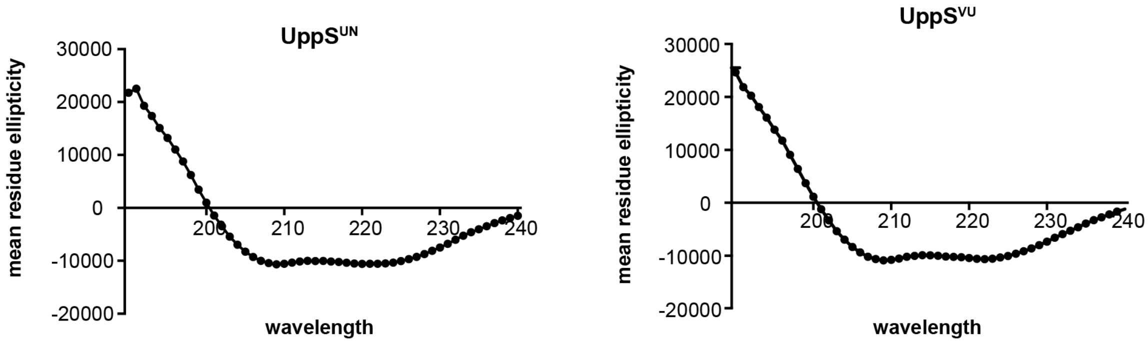

Helix 1	Helix 2	Strand 1	Strand 2	Turns	Unordered	Total
0.22	0.14	0.1	0.07	0.16	0.31	1

Helix 1	Helix 2	Strand 1	Strand 2	Turns	Unordered	Total
0.21	0.14	0.09	0.07	0.16	0.32	0.99

C

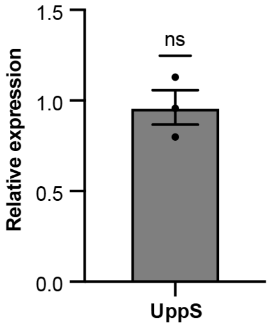

Figure S3. Dilution spotting of 17978UN wild-type, Δ*mlaF,* and Δ*mlaF* suppressor strains. Overnight cultures were serially diluted in PBS before plating on an LB agar plate with or without 0.01% SDS + 0.125 mM EDTA for overnight incubation. Experiments were repeated two independent times (total n=4) with similar results.

Figure S4. 17978VU and 17978UN capsule, peptidoglycan, and LOS staining.

(A) The peptidoglycan of 17978VU and 17978UN wild-type, Δ*mlaF, and* Δ*mlaF* with the opposite *uppS* allele was stained with NADA-green (3-[(7-Nitro-2,1,3-benzoxadiazol-4-yl)amino]-D-alanine hydrochloride). Scale bar = 5 μm. Images are representative of two biological replicates from one experiment.

(B) 17978VU and 17978UN wild-type and Δ*mlaF* strains were subjected to Maneval's capsule stain. Scale bar = 5 μm. Experiments were repeated three independent times (total n=5) with similar results.

(C) Raw image of Alcian blue stained capsular polysaccharide. Experiments were repeated three independent times (total n=7) with similar results.

(D) Raw image of silver-stained LOS gel. Experiments were repeated three independent times (total n=7) with similar results.

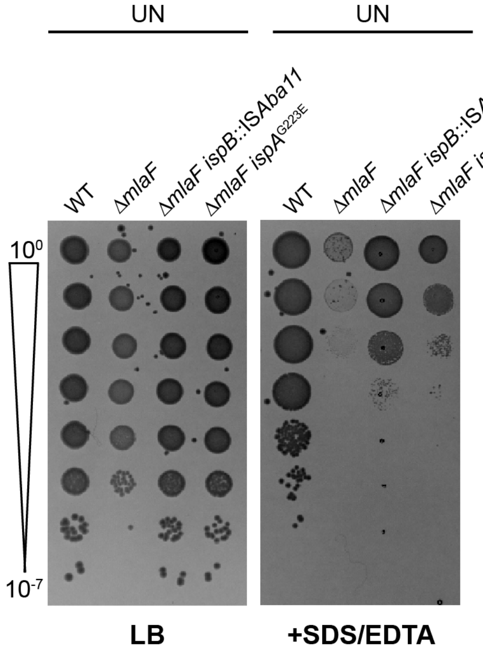

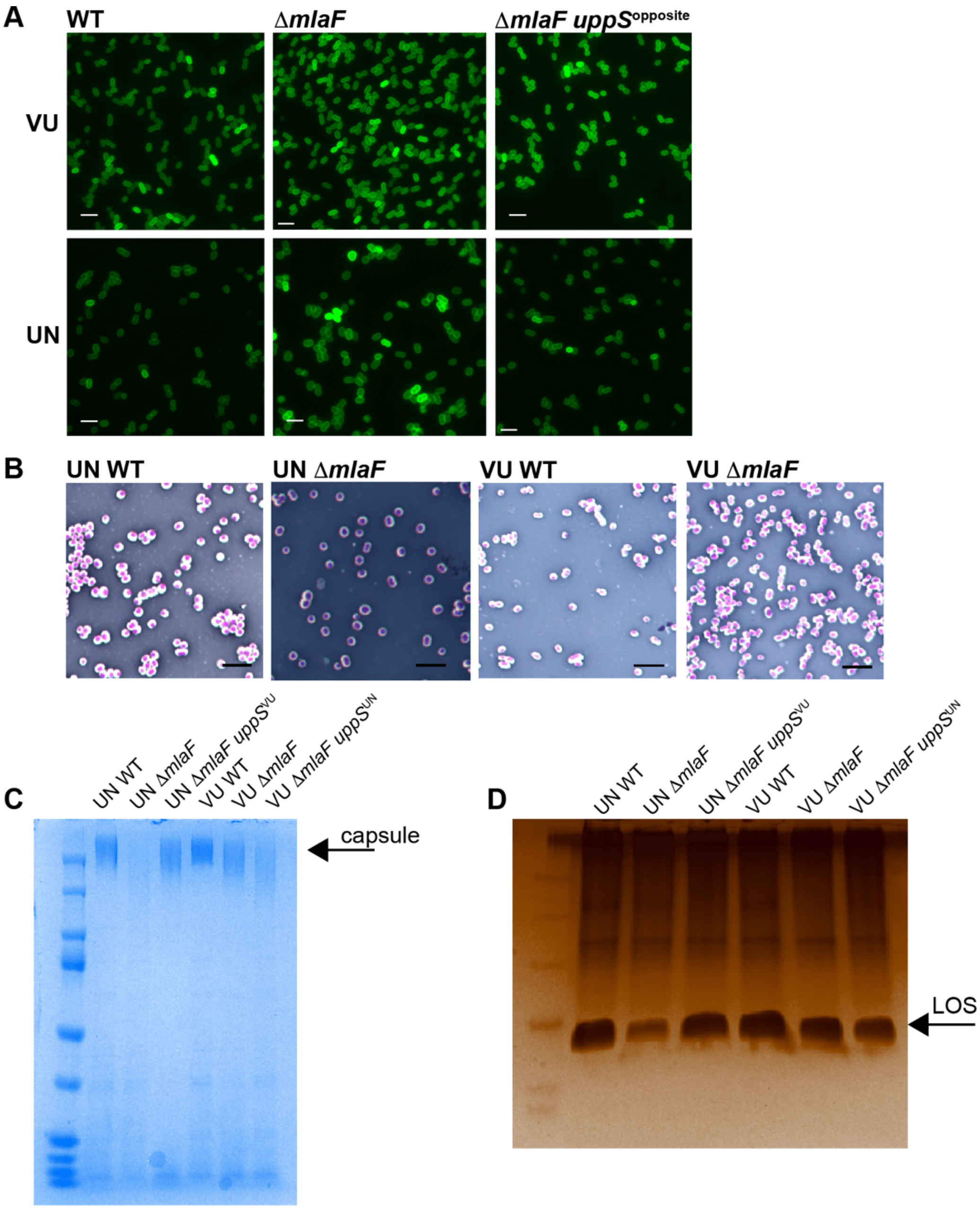
A
WT
ΔmlaF
ΔmlaF uppS^opposite
VU
UN
B
UN WT
UN ΔmlaF
VU WT
VU ΔmlaF
C
UN WT
UN ΔmlaF
UN ΔmlaF uppS^VU
VU WT
VU ΔmlaF
VU ΔmlaF uppS^UN
capsule
D
UN WT
UN ΔmlaF
UN ΔmlaF uppS^VU
VU WT
VU ΔmlaF
VU ΔmlaF uppS^UN
LOS

Figure S5. Antibiotic susceptibility, lysozyme sensitivity, and bacterial burdens from a lung infection model.
(A-B) Antimicrobial susceptibility of *A. baumannii* ATCC 17978VU and 17978UN wild-type and mutant strains was determined by a disk-diffusion assay and measuring the zone of clearance. Data are representative of 3 experiments. n=3, data are mean ± SEM. Significance is by one-way ANOVA with Tukey's multiple comparisons.
(C) Growth curve in LB with and without lysozyme at 1 mg/mL. n=3, data are means +/- SEM.
(D-G) Bacterial CFU were enumerated 48 h after intranasal inoculation. n=5, medians are shown, and significance was determined by a Mann-Whitney test, * $p < 0.05$

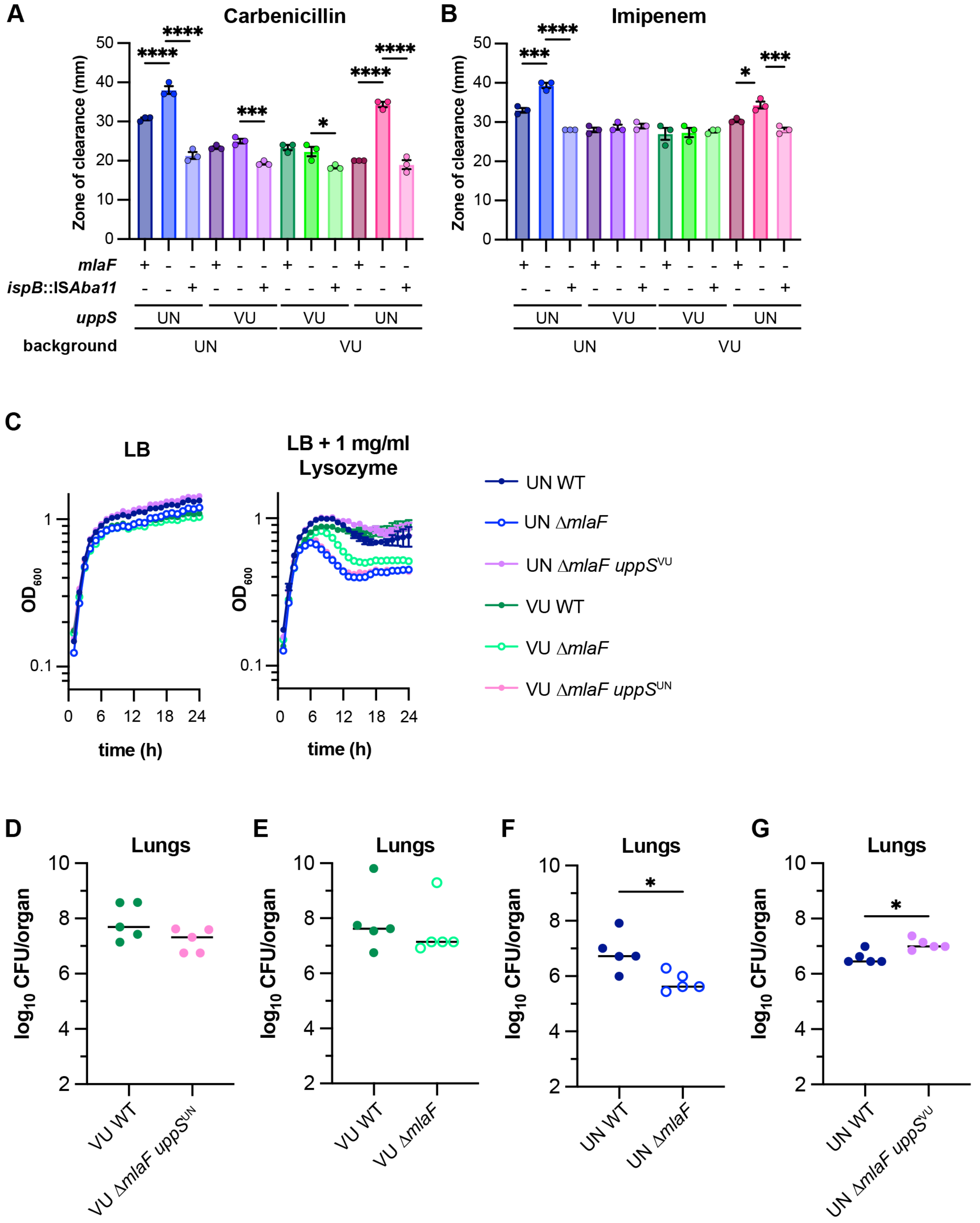
A
Carbenicillin
Zone of clearance (mm)
mlaF
ispB::ISAba11
uppS
background
B
Imipenem
C
LB
LB + 1 mg/ml Lysozyme
OD600
time (h)
UN WT
UN ΔmlaF
UN ΔmlaF uppSVU
VU WT
VU ΔmlaF
VU ΔmlaF uppSUN
D
E
F
G
Lungs
log10 CFU/organ
VU WT
VU ΔmlaF uppSUN
VU ΔmlaF
UN WT
UN ΔmlaF
UN ΔmlaF uppSVU

Table S1. Strains

Strain	Source	Identifier
A. baumannii 17978UN (WT)	ATCC	LP303
A. baumannii 17978VU (WT)	ATCC	LP486
A. baumannii 17978UN Δ*mlaF*(*A1S_3103*)::kan	Palmer et. al. 2020 (1)	LP39
A. baumannii 17978VU Δ*mlaF*::kan	This manuscript	LP472
A. baumannii 17978UN Δ*mlaF*::kan, *ACX60_03855*::IS*Aba11*	Palmer et al. 2020 (1)	LP338
A. baumannii 17978VU Δ*mlaF*::kan, *ACX60_03855*::IS*Aba11*	This manuscript	LP485
A. baumannii 17978VU, *obgE* I258	This manuscript	LP477
A. baumannii 17978VU Δ*mlaF*::kan, *obgE* I258	This manuscript	LP484
A. baumannii 17978VU, *lptD* V799	This manuscript	LP521
A. baumannii 17978VU Δ*mlaF*::kan, *lptD* V799	This manuscript	LP522
A. baumannii 17978UN, *uppS* M78	This manuscript	LP628
A. baumannii 17978VU, *uppS T*78	This manuscript	LP523
A. baumannii 17978UN Δ*mlaF*::kan, *uppS* M78	This manuscript	LP629
A. baumannii 17978VU Δ*mlaF*::kan, *uppS T*78	This manuscript	LP540
A. baumannii 17978UN Δ*mlaF*::kan, *ACX60_03855*::IS*Aba11*, *uppS* M78	This manuscript	LP640
A. baumannii 17978VU Δ*mlaF*::kan, *ACX60_03855*::IS*Aba11*, *uppS T*78	This manuscript	LP622
A. baumannii 17978UN Δ*mlaF*::kan, *uppS T*78, *ispA* (*ACX60_03710) G223E*	This manuscript	LP798

Table S2. Plasmids

Use or Phenotype	Source	Name
KnR; mobilization helper plasmid	Figurski and Helinski, 1979 (2)	pRK2013
CarbR; allelic exchange vector with sucrose sensitivity	Hoang et al., 1998 (3)	pFLP2
Contains an FRT-flanked kanamycin resistance gene	Datsenko and Wanner, 2000 (4)	pKD4
CarbR; allelic exchange vector to delete *mlaF*; pFLP2-Δ*mlaF*::Kn	Palmer et. al. 2020 (1)	pLDP8
CarbR; allelic exchange vector to swap *obgE* I258 allele to *obgE* N258; pFLP2-*obgE**	This manuscript	pLDP84
CarbR; allelic exchange vector to swap *obgE* N258 allele to *obgE* I258; pFLP2-*obgE*	This manuscript	pLDP88
CarbR; allelic exchange vector to swap *lptD* allele to *lptD* V799; pFLP2-*lptD* V799	This manuscript	pLDP104
CarbR; allelic exchange vector to swap *lptD* allele to *lptD F*799; pFLP2-*lptD* F799	This manuscript	pLDP105
CarbR; allelic exchange vector to swap *uppS* allele to *uppS* M78; pFLP2-*uppS*M78	This manuscript	pLDP106
CarbR; allelic exchange vector to swap *uppS* allele to *uppS* T78; pFLP2-*uppS*T78	This manuscript	pLDP107
CarbR; allelic exchange vector to regenerate the ispA* suppressor mutation (*ACX60_03710 G223E)*; pFLP2-ispA*	This manuscript	pLDP187
CarbR; $UppS^{VU}$ expression vector for protein purification	This manuscript	pLDP192
CarbR; $UppS^{UN}$ expression vector for protein purification	This manuscript	pLDP193

Table S3. Oligonucleotides

Name	Sequence	Source	Name
pFLP2 fwd	TGAACGGCAGGTATATGTGATGGG	IDT (Coralville, IA)	LP52
pFLP2 rev	CCATGATTACGAATTCGAGC	IDT (Coralville, IA)	LP54
pKNOCKseq_fwd	GCGCTTTTGAAGCTAATTCG	IDT (Coralville, IA)	LP354
pKNOCKseq rev	ATTTCACTTATCTGGTTGGCC	IDT (Coralville, IA)	LP355
pFLP2_ObgE_fwd	AAAGGATCGATCCTCTAGAGTGTTCAT CCTTAAAGCTAATTTG	IDT (Coralville, IA)	HN1
pFLP2_ObgE_rev	TACGAATTCGAGCTCGGTACCTGTTAA AATAATTAATAAATCAGCATGTAC	IDT (Coralville, IA)	HN2
Up_ObgE_fwd	CTCTGTTCAGCACACTAAAGC	IDT (Coralville, IA)	HN3
Dn_ObgE_fwd	CACCACCACCAGCCATTTC	IDT (Coralville, IA)	HN4
ObgE*_seq_fwd	GTACCCGTTGGTACAACAATTG	IDT (Coralville, IA)	HN5
ObgE*_seq_rev	GATTCGTTTACACTCACTGAGC	IDT (Coralville, IA)	HN6
ObgE_MutSite_fwd	GTTCAGATCCGGCCCATAA	IDT (Coralville, IA)	HN7
ObgE*_MutSite_fwd	GTTCAGATCCGGCCCATAT	IDT (Coralville, IA)	HN8
pFLP2_LptD_FWD	GGTTAAAAAGGATCGATCCTCTAGAG GATCATTCTGCACAAGAAAGTAGCGG TACGCGTA	IDT (Coralville, IA)	HN23
pFLP2_LptD_rev	TGACCATGATTACGAATTCGAGCTCG GTACAATCTTCACCCGCTTTTAATCGC TTGTAAA	IDT (Coralville, IA)	HN24
pFLP2_UppS_fwd	GATCGATCCTCTAGAGGATCTTTAACG GCAATTTGCTTATG	IDT (Coralville, IA)	HN25
pFLP2_UppS_rev	TACGAATTCGAGCTCGGTACTTACCG AATTTACGGCCTAC	IDT (Coralville, IA)	HN26
Up_LptD_fwd	GATGCAGACAAGCCATATG	IDT (Coralville, IA)	HN27
Dn_LptD_rev	CCTACAGGAATTTCCTGC	IDT (Coralville, IA)	HN28
Up_UppS_fwd	GAAAGCGACCAAAGTAGAC	IDT (Coralville, IA)	HN29
Dn_UppS_rev	CAAGAATAGAAGTTGTTGAACCC	IDT (Coralville, IA)	HN30
UppS_T78mutsite_fwd	CAATATGAAGTCGATCTGCTTAC	IDT (Coralville, IA)	HN31
UppS_M78mutsite_fwd	CAATATGAAGTCGATCTGCTTAT	IDT (Coralville, IA)	HN32
UppS_seq_fwd	CTATTACTGCTGATTCAATCCG	IDT (Coralville, IA)	HN33
LptD_F799mutsite_fwd	CGTCTTTACTTGAAAATCGCT	IDT (Coralville, IA)	HN34
LptD_V799mutsite_fwd	GTCTTTACTTGAAAATCGCG	IDT (Coralville, IA)	HN35
LptD_seq_fwd	GCTATTATTTTGAAGATCGCC	IDT (Coralville, IA)	HN36
pFLP2-ispA*_f	AAAAGGATCGATCCTCTAGAGGATCG GCTGGTGGTGGAGAACG	IDT (Coralville, IA)	HN85
pFLP2-ispA*_r	ATGACCATGATTACGAATTCGAGCTGC AATTTTAGAAGCAGATGGTCGTT	IDT (Coralville, IA)	HN86
dn_dn_ispA_r	CAACGTCGTGCTACAGTTAC	IDT (Coralville, IA)	HN87
up_up_ispA*_f	CAGCAGCATTAGCACGTTTTG	IDT (Coralville, IA)	HN88
ispA*seq_f	CTTGTATGGACAATGATTTGCTTC	IDT (Coralville, IA)	HN89
pET15b-UppS-fwd	TTCGGGCTTTGTTAGCAGCCGGATCT TATAATTTCTCGATTTTCTCTTGC	IDT (Coralville, IA)	HN94

pET15b-UppS-rev	GGCCTGGTGCCGCGCGGCAGCCATA TGACCGATTCAGAAGAGTAT	IDT (Coralville, IA)	HN95
UppS-qPCR-fwd	GATGGGCACAGAGAGGGTAA	IDT (Coralville, IA	HN215
UppS-qPCR-rev	ACGATCCCCTATAAAGCGCA	IDT (Coralville, IA	HN216

Supplemental methods

Maneval stain for capsule

Strains were streaked to LB and incubated overnight at 37°C. A single colony was resuspended in 10 μL of 1% Congo Red and spread across a microscope slide. Slides were flooded with Maneval Stain (acid fuchsin, glacial acetic acid, iron(III) chloride)(5) for 5 minutes before gentle washing and imaging at 60X on a Keyence BZ-X Inverted Fluorescence microscope (Itasca, IL).

NADA-green peptidoglycan stain

3-[(7-Nitro-2,1,3-benzoxadiazol-4-yl)amino]-D-alanine hydrochloride (NADA) was used as previously described (6). Cultures were grown overnight at 37°C. The following morning, cells were subcultured into fresh media and grown to mid-log phase with an OD_{600} of 0.5. 1 mL of OD_{600} 0.5 cells were pelleted, washed in LB, and resuspended in 1 mL fresh LB. NADA-green (ThermoFisher, Waltham, MA) was added to the resuspended cells to a final concentration of 30 μM and incubated for 30 minutes at 37°C. Cells were then fixed in 1.6% paraformaldehyde and plated on an agar pad. Imaging was performed at 60X using a Keyence BZ-X Inverted Fluorescence microscope (Itasca, IL).

Circular dichroism

$UppS^{VU}$ and $UppS^{UN}$ were prepared to 0.1 mg/mL in circular dichroism (CD) buffer (10 mM sodium phosphate buffer, pH 8.0, with 10% glycerol). CD spectra were collected by the UIC BioPhysics Core from a 0.1 mg/mL sample in a 1 mm cuvette from 190 nm to 240 nm on a Jasco 815. Protein secondary structure was determined by the software CDSSTR (7, 8).

Transcript analysis

RNA was isolated from three biological replicates of ATCC 17978VU and 17978UN wild-type strains using the RNeasy kit (Qiagen, Hilden, Germany). Overnight cultures were pelleted,

incubated with ice cold 1:1 acetone:ethanol before pelleting again. Cells were resuspended in RNase-free TE buffer pH 8.0 and transferred to RNase-free bullet blender tubes before blending for 3 minutes and continuing to the RNeasy Kit manufacturer protocol. Isolated RNA was depleted of contaminating DNA using the TURBO DNase kit (Ambion, Austin, Texas) per manufacturer's instructions. cDNA was generated from the RNA by combining RNA with random hexamers and incubating at 70°C for 5 minutes. The reverse transcriptase, RNase inhibitor, dNTPs, and reverse transcriptase buffer (Promega, Madison, WI) were added to the RNA and incubated for 1 hr at 37°C. RT-qPCR was performed using GoTaq qPCR mix (Promega, Madison, WI) and measured with a CFX connect Real-Time System (Bio-rad, Hercules, CA).

Variant protein tree construction – genome filtering and de-duplication

To acquire genomes for analysis, 7431 summaries of *Acinetobacter baumannii* reference assemblies (assembly accessions starting with "GCF_") were downloaded from NCBI datasets using the datasets command line interface tool. Genomes with contig N50 scores in the bottom 20% were removed from the assemblies, resulting in a total of 5945 genomes. Since there were many closely related genomes, a deduplication process was carried out based on Average Nucleotide Identity (ANI) values estimated using Mash distances generated by Mash version 2.34 (9). Each genome was represented by a compressed min-Hash sketch, and pairwise distances between sketches were used to construct a standard distance matrix. To perform the deduplication, a custom Python script iterated through each genome in the distance matrix, comparing its distances to other genomes. A chosen threshold Mash distance (t_{dist}) of 0.006 was used. If the pairwise distance between two genomes was less than t_{dist}, the genome with the lower N50 score was discarded. This step was performed recursively until all pairwise distances were greater than t_{dist}, ensuring that only unique genomes remained in the dataset. Ten

assemblies were identified as outliers due to their high average Mash distance to all other genomes and were excluded. Scripts for downloading genomes, creating Mash sketches and distance matrices, and dereplicating genomes and removing outliers are available at https://github.com/JonWinkelman/genome_deduplication.

Variant protein tree construction – identification of orthologs in A. baumannii

We utilized OrthoFinder (10, 11) version 2.5.4 with default settings to determine orthologous relationships between genes in *A. baumannii* proteomes. A total of 233 proteomes from filtered and de-duplicated *Acinetobacter* genomes were included in the analysis. These genomes include three outgroups*, A. baylyi*, *A. gyllenbergii* and *A. colistiniresistens* that were used to root the species tree. OrthoFinder computed hierarchical orthologous groups (HOGs) for each internal node in the species tree. These HOGs consist of proteins descended from a single gene in the ancestral species corresponding to the respective internal node. For this study, we focused on analyzing HOGs associated with the species tree node representing the last common ancestor of all *A. baumannii*. OrthoFinder computes HOGs for each internal node of the species tree. Each HOG contains all genes that descended from a single gene in ancestral species represented by the internal node. In this analysis we analyzed HOGs for the species tree node representing the last common ancestor of all *A. baumannii.* A custom python dash application was used to explore results from OrthoFinder, and iTOL was used to annotate trees for figures (12). The proteomes and alleles depicted are listed in Table S4.

Variant protein tree construction – Manual identification of orthologs in A. baumannii

When working with three specific genes, OrthoFinder search for orthologs didn't yield results across all species. In cases where OrthoFinder didn't locate an ortholog, we adopted an alternative approach. Specifically, we investigated the presence or absence of neighboring genes. For instance, if an ortholog for the *Acinetobacter baumannii* 17978-mff gene *ACX60_11495* was detected in a strain, orthologs to all other genes in its operon were found.

Conversely, when this ortholog was absent, none of the operon's other genes were found, suggesting that this gene and its operon were indeed absent from the genome.

In another instance involving the *Acinetobacter baumannii* 17978-mff gene *ACX60_05080*, OrthoFinder failed to identify orthologs in two strains. In this case, we observed adjacent orthologs and identified a potential ortholog with an unassigned HOG and more than 95% sequence identity to ACX60_05080. These findings strongly suggested that this identified potential ortholog was indeed an orthologous gene. Jupyter notebooks for downloading genomes, creating Mash sketches and distance matrices, dereplicating genomes, removing outliers, processing OrthoFinder results and creating figures are available at https://github.com/JonWinkelman/Palmer_baumannii_UNvVUprevalence.git.

Supplemental references

1. Palmer LD, Minor KE, Mettlach JA, Rivera ES, Boyd KL, Caprioli RM, Spraggins JM, Dalebroux ZD, Skaar EP. 2020. Modulating isoprenoid biosynthesis increases lipooligosaccharides and restores *Acinetobacter baumannii* resistance to host and antibiotic stress. Cell Rep 32:108129.

2. Figurski DH, Helinski DR. 1979. Replication of an origin-containing derivative of plasmid RK2 dependent on a plasmid function provided in trans. Proc Natl Acad Sci U S A 76:1648–1652.

3. Hoang TT, Karkhoff-Schweizer RR, Kutchma AJ, Schweizer HP. 1998. A broad-host-range Flp-FRT recombination system for site-specific excision of chromosomally-located DNA sequences: application for isolation of unmarked *Pseudomonas aeruginosa* mutants. Gene 212:77–86.

4. Datsenko KA, Wanner BL. 2000. One-step inactivation of chromosomal genes in *Escherichia coli* K-12 using PCR products. Proc Natl Acad Sci U S A 97:6640–6645.

5. Maneval WE. 1941. Staining bacteria and yeasts with acid dyes. Stain Technol 16:13–19.

6. Kang KN, Kazi MI, Biboy J, Gray J, Bovermann H, Ausman J, Boutte CC, Vollmer W, Boll JM. 2021. Septal class A penicillin-binding protein activity and LD-transpeptidases mediate selection of colistin-resistant lipooligosaccharide-deficient *Acinetobacter baumannii*. mBio 12:10.1128/mbio.02185-20.

7. Manavalan P, Johnson WC. 1987. Variable selection method improves the prediction of protein secondary structure from circular dichroism spectra. Anal Biochem 167:76–85.

8. Compton LA, Johnson WC. 1986. Analysis of protein circular dichroism spectra for secondary structure using a simple matrix multiplication. Anal Biochem 155:155–167.

9. Ondov BD, Treangen TJ, Melsted P, Mallonee AB, Bergman NH, Koren S, Phillippy AM. 2016. Mash: fast genome and metagenome distance estimation using MinHash. Genome Biol 17:132.

10. Emms DM, Kelly S. 2015. OrthoFinder: solving fundamental biases in whole genome comparisons dramatically improves orthogroup inference accuracy. Genome Biol 16:157.

11. Emms DM, Kelly S. 2019. OrthoFinder: phylogenetic orthology inference for comparative genomics. Genome Biol 20:238.

12. Letunic I, Bork P. 2007. Interactive Tree Of Life (iTOL): an online tool for phylogenetic tree display and annotation. Bioinforma Oxf Engl 23:127–128.
